# *Iam hiQ*—a novel pair of accuracy indices for imputed genotypes

**DOI:** 10.1186/s12859-022-04568-3

**Published:** 2022-01-24

**Authors:** Albert Rosenberger, Viola Tozzi, Heike Bickeböller, the INTEGRAL-ILCCO consortium, Rayjean J. Hung, David C. Christiani, Neil E. Caporaso, Geoffrey Liu, Stig E. Bojesen, Loic Le Marchand, Demetrios Albanes, Melinda C. Aldrich, Adonina Tardon, Guillermo Fernández-Tardón, Gad Rennert, John K. Field, Mike Davies, Triantafillos Liloglou, Lambertus A. Kiemeney, Philip Lazarus, Aage Haugen, Shanbeh Zienolddiny, Stephen Lam, Matthew B. Schabath, Angeline S. Andrew, Eric J. Duell, Susanne M. Arnold, Hans Brunnström, Olle Melander, Gary E. Goodman, Chu Chen, Jennifer A. Doherty, Marion Dawn Teare, Angela Cox, Penella J. Woll, Angela Risch, Thomas R. Muley, Mikael Johansson, Paul Brennan, Maria Teresa Landi, Sanjay S. Shete, Christopher I. Amos

**Affiliations:** 1 Department of Genetic Epidemiology, University Medical Center, Georg-August-University Göttingen, Göttingen, Germany; 2 Institut für Genetische Epidemiologie, Universitätsmedizin Göttingen, Humboldtallee 32, 37073 Göttingen, Germany; 3 Lunenfeld-Tanenbaum Research Institute, Sinai Health System, University of Toronto, Toronto, ON Canada; 4 Department of Environmental Health, Harvard T.H. Chan School of Public Health and Massachusetts General Hospital/Harvard Medical School, Boston, MA USA; 5 Division of Cancer Epidemiology and Genetics, National Cancer Institute, US National Institutes of Health, Bethesda, MD USA; 6 Medical Oncology and Medical Biophysics, Princess Margaret Cancer Centre, Toronto, ON Canada; 7 Medicine and Epidemiology, Dalla Lana School of Public Health, University of Toronto, Toronto, ON Canada; 8 Department of Clinical Biochemistry, Herlev and Gentofte Hospital, Copenhagen University Hospital, Copenhagen, Denmark; 9 Faculty of Health and Medical Sciences, University of Copenhagen, Copenhagen, Denmark; 10 Copenhagen General Population Study, Herlev and Gentofte Hospital, Copenhagen, Denmark; 11 Epidemiology Program, University of Hawaii Cancer Center, Honolulu, HI USA; 12 Department of Thoracic Surgery, Division of Epidemiology, Vanderbilt University Medical Center, Nashville, TN USA; 13 ISPA and CIBERESP, Faculty of Medicine, University of Oviedo, Oviedo, Spain; 14 Clalit National Cancer Control Center at Carmel Medical Center and Technion Faculty of Medicine, Haifa, Israel; 15 Roy Castle Lung Cancer Research Programme, Department of Molecular and Clinical Cancer Medicine, The University of Liverpool, Liverpool, UK; 16 Departments of Health Evidence and Urology, Radboud University Medical Center, Nijmegen, The Netherlands; 17 Department of Pharmaceutical Sciences, College of Pharmacy, Washington State University, Spokane, WA USA; 18 National Institute of Occupational Health, Oslo, Norway; 19 British Columbia Cancer Agency, Vancouver, BC Canada; 20 Department of Cancer Epidemiology, H. Lee Moffitt Cancer Center and Research Institute, Tampa, FL USA; 21 Department of Epidemiology, Geisel School of Medicine, Dartmouth College, Hanover, NH USA; 22 Unit of Biomarkers and Susceptibility, Oncology Data Analytics Program, Catalan Institute of Oncology (ICO), Bellvitge Biomedical Research Institute (IDIBELL), Barcelona, Spain; 23 Markey Cancer Center, University of Kentucky, Lexington, KY USA; 24 Department of Clinical Sciences, Laboratory Medicine Region Skåne, Lund University, Pathology, Lund, Sweden; 25 Department of Clinical Sciences, Skåne University Hospital, Internal Medicine, Lund University, Malmö, Sweden; 26 Swedish Medical Group, Seattle, WA USA; 27 Program in Epidemiology, Fred Hutchinson Cancer Research Center, Seattle, WA USA; 28 Department of Population Health Sciences, Huntsman Cancer Institute, University of Utah, Salt Lake City, Utha, USA; 29 Institute of Health and Society, Newcastle University, Newcastle upon Tyne, UK; 30 Department of Oncology and Metabolism, School of Health and Related Research, University of Sheffield, Sheffield, UK; 31 Academic Unit of Clinical Oncology, University of Sheffield, Weston Park Hospital, Sheffield, UK; 32 University of Salzburg and Cancer Cluster Salzburg, Salzburg, Austria; 33 Thoraxklinik, University of Heidelberg, Germany and German Center for Lung Research (DZL), Heidelberg, Germany; 34 Department of Radiation Sciences, Umeå University, Umeå, Sweden; 35 International Agency for Research on Cancer, World Health Organization, Lyon, France; 36 Department of Biostatistics, Division of Basic Sciences, The University of Texas MD Anderson Cancer Center, Houston, TX USA; 37 Dan L Duncan Comprehensive Cancer Center, Baylor College of Medicine, Houston, TX USA

**Keywords:** GWAS, High-throughput genotyping, Genotype imputation, Accuracy measures

## Abstract

**Background:**

Imputation of untyped markers is a standard tool in genome-wide association studies to close the gap between directly genotyped and other known DNA variants. However, high accuracy with which genotypes are imputed is fundamental. Several accuracy measures have been proposed and some are implemented in imputation software, unfortunately diversely across platforms. In the present paper, we introduce *Iam* *hiQ*, an independent pair of accuracy measures that can be applied to dosage files, the output of all imputation software. *Iam* (*imputation accuracy measure*) quantifies the average amount of individual-specific versus population-specific genotype information in a linear manner. *hiQ (heterogeneity in quantities of dosages)* addresses the inter-individual heterogeneity between dosages of a marker across the sample at hand.

**Results:**

Applying both measures to a large case–control sample of the International Lung Cancer Consortium (ILCCO), comprising 27,065 individuals, we found meaningful thresholds for *Iam* and *hiQ* suitable to classify markers of poor accuracy. We demonstrate how Manhattan-like plots and moving averages of *Iam* and *hiQ* can be useful to identify regions enriched with less accurate imputed markers, whereas these regions would by missed when applying the accuracy measure *info *(implemented in IMPUTE2).

**Conclusion:**

We recommend using *Iam hiQ* additional to other accuracy scores for variant filtering before stepping into the analysis of imputed GWAS data.

**Supplementary Information:**

The online version contains supplementary material available at 10.1186/s12859-022-04568-3.

## Background

To date information of more than 660 million reference single nucleotide polymorphisms (refSNPs) and 5.9 million regions with structural variation (SV) on the human DNA are known and stored in the publicly available databases, like dbSNP [1]. To identify those genetic variants, that are associated with common human diseases, genome-wide association studies (GWAS) can be conducted. Usually, commercial single nucleotide polymorphism (SNP) microarrays are used to carry out genotyping of DNA samples for these studies. There are two predominant companies for high throughput genotyping arrays, Thermo Fisher Scientific Inc., Santa Clara, CA (Affymetrix™) and Illumina Inc., San Diego, CA. The underlying chemistry differs but both array types can be used to ascertain genotypes in a similar fashion [2]. In contrast to the more expensive and error prone new generation sequencing technologies, the number of genotyped variants ranges from 300,000 to 4 million. Array-based markers are supposed to tag the genomic region in their vicinity, but represent only a small proportion of all known DNA variants. Furthermore, these variants are not a random selection but have been chosen according to criteria such as minor allele frequency (MAF), location in exons or blocks of linkage disequilibrium or putative associations with certain disease.

Imputation methods and strategies have been developed and are now a standard tool in GWAS to close the gap between genotyped and existing DNA variants [3–5]. These methods transfer information of DNA structure from one or several reference panels with high marker density (e.g. 1000 Genomes Project phase 3 [6] or Haplotype Reference Consortium (HRC) [7]) to the genotyped study samples [4]. Most imputation methods estimate a-posteriori genotype probabilities (referred to as *dosages*, ranging from 0 to 1) for each untyped variant and each individual in the sample of interest. The resulting increase of variant density in the study sample improves the genomic coverage and can increase the power to identify genomic variants associated with a trait [8]. Imputation further has the potential that an identified associated marker is located closer to a true risk locus; it facilitates fine mapping of causal variants and is essential for meta-analyses of GWAS, particularly when different genotyping arrays have been used for multiple studies [9]. However, imputation requires advanced statistical methods for data analysis and may introduce extra uncertainty in interpreting findings. Further, only DNA variants that have previously been genotyped in the used reference panel can be imputed [4, 10].

Imputation methods based on linkage disequilibrium (LD) information (e.g. fastPHASE [11]; MaCH [12, 13]; Beagle [14]; IMPUTE2 [15]) and are suitable for samples of independent individuals, as in case–control studies. Other methods use pedigree and linkage information (e.g. F-Impute [16]; α-Impute [3, 17]), and are therefore suitable for related individuals.

### Known accuracy measures

It is important to evaluate the quality of imputation, e.g. to exclude poorly imputed variants from statistical analysis. Several quality indices have been developed and are routinely applied [4, 5, 18]. These comprise *inter alia* the squared correlation *r*^2^ between the true and imputed dose of an allele across all imputed samples (MaCH *r*^2^, Minimac or Beagle *r*^2^) or IMPUTE2’s *info*.

All *r*^2^ measures can be derived from a-posteriori allele probabilities without knowledge of the true allele dose, but only if the allele probabilities are well calibrated and MAF is not too low. The power of an allelic test with *N* samples and imputed alleles is approximately equal to the power of the same test with *r*^2^*N* samples and known alleles, in case of a binary trait. Differences among the known *r*^2^ measures are discussed elsewhere [4]. The commonly used *info* is defined as the proportion of statistical information on the population allele frequency in the imputed genotypes, relative to “known” genotypes [5]. If the Hardy–Weinberg disequilibrium (HWE) holds, *info* equalizes to Minimacs *r*^2^. Hence, *r*^2^*-*based measures and *info* are directly related to the power of statistical test of a marker x trait association.

In general, both metrics have preferable characteristics if the a-posteriori genotype probabilities (dosages) are accurately calculated [18]. However, multiple factors can affect imputation accuracy, e.g. sample size, sequencing coverage and haplotype accuracy of the references panel(s), density of the genotyping array, allele frequency and poor LD between genotyped and imputed variants [4]. One can calculate these accuracy measures from dosage files. However, the standard outputs of common imputation programs (e.g. Beagle or IMPUTE2) contain different metrics. Hence, choosing an imputation program binds the user to the metrics provided, although the SNPTEST program offers the option of calculating a measure similar to that of info [19].

We propose a new pair of metrics to depict additional aspects of imputation accuracy also calculable from dosage files. First, we aim to quantify the amount of individual-specific versus population-specific genotype information in the imputed genotypes. Second, we aim to assess the heterogeneity between dosages of a marker across the sample at hand. Both measures can be used to identify markers or regions in which population-specific genetic information conceal individual-specific information and are therefore less informative for e.g. association testing. These new metrics are not intended as a competitor to established scores, but are intended to support the making of well-founded decisions in SNP filtering of imputed markers prior to an analysis or in interpretation of results after an analysis.

We calculated this pair of accuracy measures on a series of 27,065 cases and controls gathered by the International Lung Cancer Consortium (ILCCO) to find meaningful thresholds for marker exclusion and compared it with *info*, because all of the ILCCO samples had previously been imputed with IMPUTE2 applied to a standard 1000 Genomes referent panel. Further, we contrasted the usability of the new measures to *info* in some simulated data.

## Results

### Comparison of *Iam* and *hiQ*

When applying the novel indices *Iam hiQ* (as defined in the section *Novel accuracy measures*) to 517,482 SNPs types with the OncoArray, only a small portion (n = 40,678, 4‰) can be considered as imputed without doubt (*Iam* = 1 and *hiQ* = 1). For the majority of SNPs a value between 0.95 and < 1 was assigned for *hiQ* (9,760,392, ~ 94%), while only 30% (n = 3,243,272 markers) achieved such a large value with respect to *Iam*. It is worth to mention, that we assigned a reduced value for *Iam* (from 0.4 to 0*.*75) to about as many SNPs (n = 3,491,596, 33%). More details are given in Additional file 1: Table S1.

**Fig. 1:**
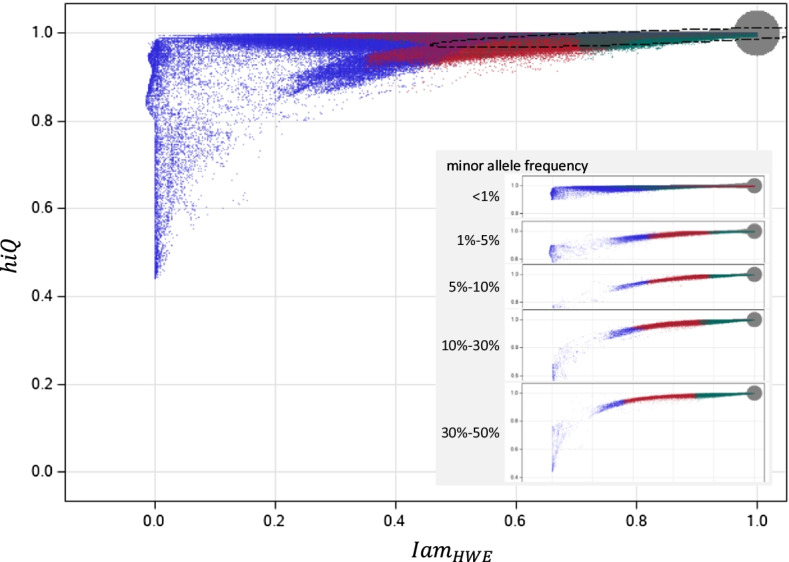
*Iam* by *hiQ.* Main panel: all markers by Iam vs. hiQ; blue dots: variants with info < 0.5; red dots: variants with 0.5 ≤ info < 0.8; 8; green dots: variants with info ≥ 0.8; dotted line: robust 99.9999999% bivariate normal random interval (assuming a two-dimensional normal distribution). The oversized grey bubble in the top right corner represents the vast majority of almost fully-informative markers with *Iam* ≥ 0.99 and *hiQ* ≥ 0.99; inserted panel: like main panel, but marker are divided according to the minor allele frequency

Both components of *Iam hiQ*, are contrasted in a bubble plot (Fig. 1). The oversized grey bubble in the top right corner represents the vast majority of almost fully-informative markers with *Iam* ≥ 0*.*99* and hiQ* ≥ 0*.*99*.* It can easily be seen that the remaining small minority of not fully accurately imputed markers take advantage of the whole theoretical range for *Iam* (even negative values). In contrast, *hiQ* always exceeds 0.4 in the sample at hand, but seems to be sensitive in markers with low values for *Iam*, whereas lower values of *hiQ* are only assigned to common markers.

### Defining thresholds for marker filtering

In order to exclude less accurately imputed variants from further analysis, one needs to define a meaningful and applicable threshold for any accuracy index. For the measure *info* threshold values like 0.8 or 0.3 have been proposed, but without sound justification [5, 20, 21]. It was even proposed to lower the threshold for *info* in very large samples, as those of the UK Biobank, and still maintain a good ability to detect associations [22].

We applied robust regression (PROG ROBUSTREG of SAS 9.4; cut-off-α = 10^–6^ for leverage points, cut-off-multiplier = 5 for outliers[23]) to estimate the expected value of *Iam* and *hiQ* and their variance–covariance matrix, assuming a hidden two-dimensional normal distribution (ignoring the upper bounds of the indices). Based on this we derived the 99.9999999% (1–10^−9^) random region (dashed line in Fig. 1) to define, very conservative and data driven, lower bounds for the two indices, limiting the probability of a false-exclusion to ~ 1/(100∙10,439,017) (one hundredth under Bonferroni correction assuming independent markers). The robust mean for *Iam* was 0.7409 that for *hiQ* was 0.9885. Restricted to common markers (MAF ≥ 0.1) we achieved similar mean values (*Iam*: 0.8101, *hiQ*: 0.9894). The lower bounds of the random region of *hiQ* were 0.9627 for all markers and 0.9673 for common markers, which is almost identical. In contrast, the lower bounds of the random region of *Iam* were 0.2553 for all markers and 0.4657 for common markers, demonstrating the influence of MAF, via HWE, on *Iam*. We decided to use the study specific thresholds of 0.47 for *Iam and *0*.*97 for *hiQ* to further classify markers of poor accuracy. Because *Iam* ranges linearly from *population informative dosages* to *fully individual informative dosages*, the threshold of ~ 0.5 indicates markers with less than ~ 50% individual-specific genotype information (in average across all samples). Such an intuitive interpretation cannot be given for *hiQ*.

For the majority of 9,094,772 (87.2%) variants, sufficient imputation accuracy was achieved, according to our defined thresholds (see Table 1). Limiting the markers to those with 0.5 < *info* ≤ 0.8 and *info* ≥ 0.8, this fraction increases to 95.1% or 99.9%, respectively. In very rare genetic variants (MAF < 1%) the fraction drops to 76.5%. In contrast, only 0.6% of variants meet neither the *Iam* nor the *hiQ* criteria. Interestingly, 1,214,620 variants (11.7%) missed only the *Iam* criteria. The fraction was larger in very rare variants (23.2%) and in variants with *info* < 0.5 (58.5%), while it was moderate in variants with 0.5 < *info* ≤ 0.8 (2.5%).

**Table 1:** Classification of markers by *Iam*_*HWE*_* and hiQ*

	***hiQ***	***Iam***_***HWE***_			
		< 0.47		≥ 0.47	
		N	%^a^	N	%^a^
All markers					
	< 0.97	59,077	0.6%	59,130	0.6%
	≥ 0.97	1,214,620	11.7%	9,094,772	87.2%
Quality defined by *info*					
* Low quality: info* < 0.8	< 0.97	59,077	1.2%	58,592	1.1%
	≥ 0.97	1,214,612	23.8%	3,777,566	73.9%
* High quality: info* ≥ 0.8	< 0.97		–	538	< 0.1%
	≥ 0.97	8	< 0.1%	5,317,206	99.9%
Minor allele frequency (MAF)					
< 1%	< 0.97	15,366	0.3%	136	< 0.1%
	≥ 0.97	1,210,505	23.2%	4,000,616	76.5%
1% to < 5%	< 0.97	13,448	0.9%	12,742	0.8%
	≥ 0.97	2,317	0.2%	1,472,328	98.1%
5% to < 10%	< 0.97	4,007	0.6%	12,117	1.8%
	≥ 0.97	7	< 0.1%	638,931	97.5%
10% to < 30%	< 0.97	8,441	0.6%	18,576	1.4%
	≥ 0.97	10	< 0.1%	1,288,714	97.9%
30% to 50%	< 0.97	9,283	1.3%	4,188	0.6%
	≥ 0.97	261	< 0.1%	721,679	98.1%
> 50%	< 0.97	8,532	0.9%	11,371	1.1%
	≥ 0.97	1,520	0.2%	972,504	97.8%

Thresholds for *Iam*_*HWE*_ (0.47) and *hiQ* (0.97) were defined according to a robust 99.9999999% bivariate normal random interval (assuming a two-dimensional normal distribution)

^a^Proportion within tabulated subgroup of markers

### Identifying markers and regions of low accuracy

**Fig. 2:**
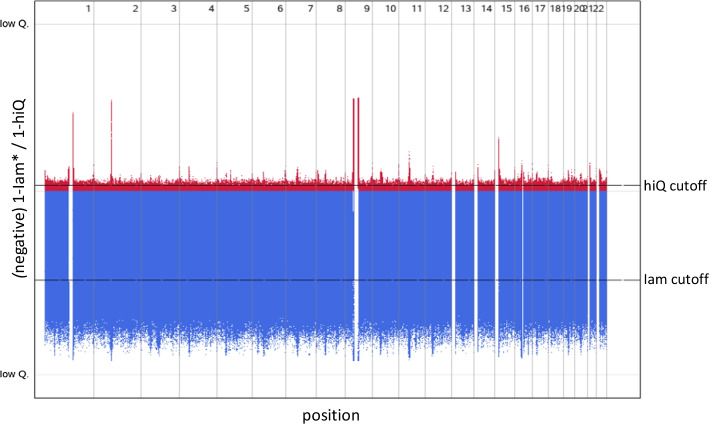
Manhattan-like-plot: *Iam hiQ.* Upper panel: $hiQ$ (low Q.: ${{hiQ}}$ = 0; high Q.: ${{hiQ}}$ = 1; Thresholds *hiQ* (cutoff = 0.97); lower panel: $Iam_{HWE}$ (low Q.: $Iam_{HWE}$ = 0; high Q.: $Iam_{HWE}$ = 1; Thresholds *Iam* cutoff = 0.47): Thresholds were defined according a robust 99.9999999% bivariate normal random interval (assuming a two-dimensional normal distribution)

Figure 2 presents the accuracy of imputed markers according to *Iam hiQ* in a Manhattan-like plot, with *Iam* given in the lower part (blue) and *hiQ* given in the upper part (red). This plot contains all 10,427,599 SNPs. Regions with massively less accurate imputation can easily be identified, especially by *hiQ* (red needles). This is for instant the case close to the centromere of chromosomes 1, 2 and 9 (accuracy by chromosome 1 is presented in Additional file 1: Figure S1). However, variants with *Iam* or *hiQ* below the defined thresholds can be found in many regions across the whole genome. Massively less accurate imputation can be found upstream the centromere, less distinct downstream the centromere and close to the telomeres, as well as around position 50K (blue icicle). Nevertheless, it is still hard to visually find regions that are enriched with less accurately imputed markers.

**Fig. 3:**
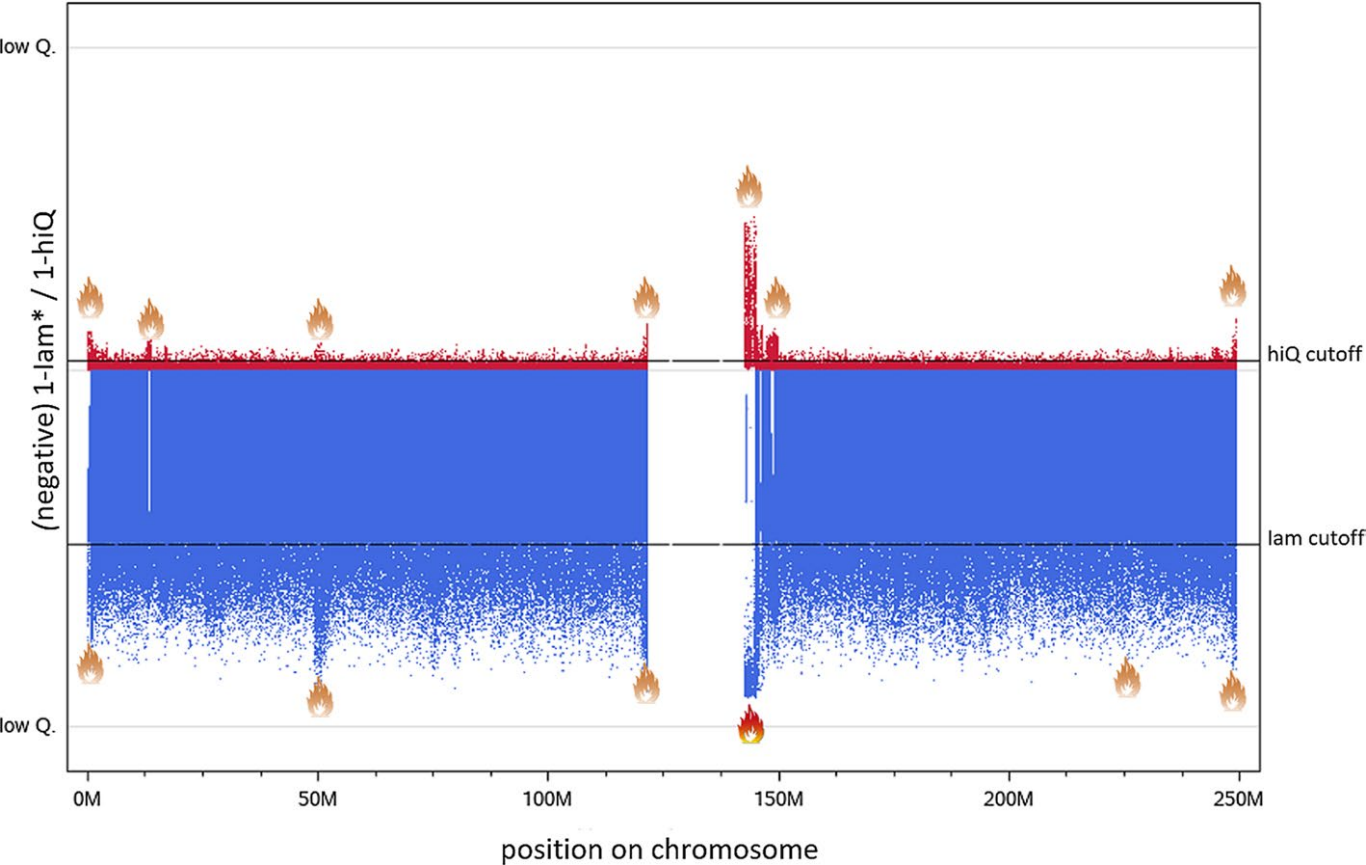
Manhattan-like-plot: *Iam hiQ*: chromosome 1. Upper panel: ${{hiQ}}$ (low Q.: ${{hiQ}}$ = 0; high Q.: ${{hiQ}}$ = 1; Thresholds *hiQ* (cutoff = 0.97); lower panel: $Iam_{HWE}$ (low Q.: $Iam_{HWE}$ = 0; high Q.: $Iam_{HWE}$ = 1; Thresholds *Iam* cutoff = 0.47): Thresholds were defined according a robust 99.9999999% bivariate normal random interval (assuming a two-dimensional normal distribution); red flames indicate “very hot” regions; orange flames indicate “hot” regions

To identify more genomic regions prone to host inaccurate markers we calculated the exponentially weighted moving averages (ewma) of *Iam* and *hiQ* (PROC EXPAND of SAS 9.4; smoothing factor 0.1) [23]. We consider variants with an *ewma* < threshold (0.47 for *Iam* and 0.97 for *hiQ*) as belonging to a “hot region” and variants with an *ewma* < threshold/2 (0.23 for *Iam* and 0.48 for *hiQ*) as belonging to a “very hot region”. Across the whole genome, we were able to identify 4,603 “hot regions” and 171 “very hot regions” according to *Iam*_*HWE*_, as well as 2,899 “hot regions” according to *hiQ*. These regions partially overlap or are interconnected. “Hot” and “very hot” *Iam*-regions contain in total 85,790 variants, only about 8‰ of all variants. “Hot” *hiQ*-regions contain in total 53,590 variants, only about 5‰ of all variants. However, about 1 out of 3 “hot” or “very hot” regions is very small and contains only one variant. In contrast, 10% of the “hot” *Iam*-regions and about 20% of either the “very hot” *Iam-* or the “hot” *hiQ*-regions contain more than 20 variants (see Additional file 1: Tables S2–S4). Some of these regions on chromosome 1 are indicated by flames in Fig. 3.

### Comparing *Iam hiQ* with *info* and *certainty*

The missing rate for *info* was about 18% and 0.6% for *certainty,* in the data set at hand. *Iam* and *hiQ* could be determined for all markers (see Additional file 1: Table S5). The correlation between *Iam* and *info* was largest (*r*^2^ = 0.944), indicating that both represent comparable information on accuracy. *hiQ* and *certainty* correlate only moderate among themselves as do the other measures (*r*^2^ < 0.5) (see Table 2). However, only every second fully-informative SNP (*Iam* = 1 and *hiQ* = 1) was assigned a value ≥ 0.8 for *info* (see Additional file 1: Table S1, red shaded points in Fig. 1), whereas this was the case for less than 2‰ of variants with reduced *Iam* (0.4 to 0*.*75). This means that *Iam* and *info* nevertheless carry different information on imputation accuracy*.*

**Table 2:** Correlation between accuracy measures

	***Iam***_*HWE*_	***hiQ***	***info***	***certainty***
*Iam*_*HWE*_	–	0.684	0.944	0.484
*hiQ*	0.405	–	0.367	0.156
*info*	0.976	0.686	–	0.050
*certainty*	0.305	0.335	0.051	–

Right upper triangle: Pearson’s correlation coefficient, left lower triangle: Spearman’ rank correlation coefficient

Figures for a visual comparison of *Iam* and *info* are included in the Additional file 1: Figures S2 and S3. These clearly show that *info* is less suitable for mapping regions enriched with less accurately imputed genotypes, genome-wide and chromosome-wide.

## Usability

We also investigated the usability of the proposed indices in contrast to *info* by simulation. Usability was considered in terms of discrimination between sufficient and insufficient imputation, rather than in terms of validity of imputation because validity is a characteristic of the imputation routine (e.g. IMPUTE2).

Eight scenarios consisting of two common genotyped tagSNPs flanking one intermediate marker for imputation were defined, differing from each other by the underlying haplotype structure, MAF and LD-patterns were defined. Two scenarios each form a pair (a scene), consisting of a scenario in which the missing marker can be imputed sufficiently/better and one scenario in which the missing marker can be imputed insufficiently/worse. Imputation was performed on 100 randomly drawn samples for each scenario, and accuracy measures were calculated. The ability of an index to discriminate a sufficient from an insufficient scenario (usability) was visually inspected plotting comparative receiver operation curves (one ROC per index) for each scene, and quantified as area under der curve (AUCs) of ROCs. Details on the simulation and the results are given in the Additional file 1.

*Info* and *Iam* appear to be comparable usable in terms of discrimination between sufficient and insufficient imputation for common SNPs. However, *hiQ* seems to be superior when MAF of the imputed marker is low.

## Suggestion on how to use *Iam hiQ*

For general use, a threshold of 0.5 for *Iam* and 0.9 for *hiQ* seems reasonable to identify markers with low accuracy. However, we do not claim that this recommendation to generally optimal. All variants with values for Iam and e.g. info below the threshold value, as well as all variants with MAF < 1% and a hiQ below the threshold value should be excluded from a data analysis in order to ensure that all aspects of the imputation quality are met. This pre-analysis marker filtering can be extended to all variants in “hot” or “very hot” regions. If association results cannot be replicated across several studies, a low value of *Iam* indicates a reduced individual-specific information content, even if, for example, info and *hiQ* imply sufficient power and genotype heterogeneity.

## Discussion

Imputation is a cost-effective tool for GWAS to fill gaps of non-genotyped variants instead of whole-genome sequencing for all recruited individuals, since global coverage in genomic information of available arrays with less than 1 million SNPs not exceeds 25% [10, 24]. However, imputation accuracy matters. Several accuracy measures have been proposed and implemented in imputation software, unfortunately diverse across platforms. Das et al. [4] favour *r*^2^, the squared correlation between true and imputed allele dose, because it is tightly related to the power of an allelic test. However, they also emphasized the importance of adequate imputed samples for the r^2^accuracy.

We introduce *Iam* *hiQ*, an independent and complementary pair of accuracy measures. Other than e.g. *r*^2^, *Iam* quantifies the amount of individual-specific versus population-specific genotype information in a linear manner for each individual before averaging, while *hiQ* addresses the inter-individual heterogeneity of dosages for a marker across the sample at hand. These new measures are not intended to compete with established scores, but should complement them. We derived meaningful, but study specific thresholds for variant filtering applying *Iam hiQ* to a large case–control sample at hand. We showed how regions enriched with less accurately imputed genotypes can be identified (computationally and visually), and finally compared *Iam hiQ* to *info*, as provided by IMPUTE2. *Iam* *hiQ* is simple to interpret: $Iam_{chance}$ of 0 indicates a complete loss of genomic information for a variant. $Iam_{HWE}$ of 0 indicates a reduction to solely population-specific (not individual-specific) genomic information for a variant. $Iam_{chance/HWE}$ of 1 indicates variants for which complete individual-specific genomic information is available. $hiQ$ of 0 indicates complete inter-individual homogeneity of *dosages* across the sample. $hiQ$ of 1 indicates that statistical tests can derive all their power from heterogeneity between *dosages*.

However, it has been discussed that any imputation accuracy measures assuming HWE to calculate "expected" genotype counts can be confounded. This was demonstrated for MaCH *r*^2^ [25]. For the proposed method, HWE is solely chosen as anchor point to define pure population informative dosages. One should keep in mind that *Iam hiQ* is just a tool for quality assurance and not a data analysis module. Thus, slight violations from HWE do not compromise their use, but in case of family data caution is advised. In such cases, one can either apply *Iam*_*HWE*_ to founders only, or use *Iam*_*chance*_.

Finally, accuracy measures with non-justified thresholds, as e.g. *info*, should be applied with caution. This in mind, we derived thresholds for *Iam* *hiQ*, in contrast to other measures, from observation on a large sample and follow a traceable logic. Because its direct and linear relationship to the average amount of individual-specific genomic information contained in the *dosages* of a marker, *Iam* is easy to interpret. By this, it differs from *r*^2^, which is approximately equal to the power of the same test with *r*^2^*N* samples.

For the presented quality assurance, we calculated *Iam hiQ* for autosomes only. Extending this to the X and Y chromosome is possible, but the sex of genotyped individual and the position of the variant on the chromosome must be taken into account when calculating a correct HWE distribution. Even an ex post application of *Iam hiQ* can be useful, particular to explain whether missed replication of an observed marker-phenotype association is due to inaccurate imputation. Since the imputation accuracy of particularly rare markers tend to be low, an improved imputation of the ILCCO samples is planned on newer panels that contain more SNPS with low MAF.

## Conclusion

In summary, *Iam hiQ* is a newly proposed pair of accuracy measures for imputed genotypes. In contrast to others, it addresses directly the contents of individual-specific genotype information and the heterogeneity between dosages. It is independent of the imputation platform and can be computed for all imputed variants. We recommend using *Iam hiQ additional to other accuracy scores* for variation filtering before stepping into the analysis of imputed GWAS data.

### Availability of data and materials

A macro for SAS® 9.4 to calculate the measures *Iam*_*HWE*_, *Iam*_*chance*_ and *hiQ* for autosomal markers based on the *dosage-file* as output of IMPUTE2 is provided with the Additional file 1.

## Materials and methods

### Novel accuracy measure

In the following we will consider $m = 1\, to\,M$ markers with two alleles ($a$ and $A$) and a MAF *f*_*A*_ in the source population of the study sample consisting of *N* individuals. The three possible genotypes *aa*, *aA*, and *AA* are indicated by allele doses 0, 1 and 2 (equal to the number of minor alleles *A* of a genotype). Imputation will result in triplets of a-posteriori genotype probabilities $$\left[ {\begin{array}{*{20}c} {p_{0} } & {p_{1} } & {p_{2} } \\ \end{array} } \right]$$, referred to as dosages*,* with $\sum\nolimits_{g = 0}^{2} {p_{g} } = 1$. We assume the whole uncertainty related to genotype imputation is contained in these triplets. The allele dose of an individual *i* for an imputed marker will then be $d_{i,m} = \sum\nolimits_{i = 0}^{2} i \cdot p_{i}$ and can take any value between 0 and 2. Multi-allelic markers are assumed to be split into pseudo-two-allele variants.

### Index of individual-specific versus population-specific genotype information: *Iam*

To quantify the amount of individual-specific versus population-specific genotype information in the *dosages* of the imputed single marker *m* for a single person *i,* we first consider the following three marginal situations:

1. The triplet of dosages takes on the values $$\left[ {\begin{array}{*{20}c} 1 & 0 & 0 \\ \end{array} } \right]$$, or in a different order, if imputation is fully sufficient, when the missing genotype is unambiguously derived from the reference panel. Thus, the dosages contain fully individual-specific genotype information.
2. In contrast, if all genotypes are equally likely the dosages take on the values $$\left[ {\begin{array}{*{20}c} {1/3} & {1/3} & {1/3} \\ \end{array} } \right]$$ and imputing of the missing genotype failed (choosing a best guess genotype would be completely due to *chance*). The dosages contain no individual-specific genotype information at all.
3. If the dosages take on the values $$\left[ {\begin{array}{*{20}c} {f_{A}^{2} } & {2f_{A} \left( {1 - f_{A} } \right)} & {\left( {1 - f_{A} } \right)^{2} } \\ \end{array} } \right]$$ and hence follow HWE, imputation used solely MAF in the reference population and thus the dosages contain solely population-specific information.

To construct an index to distinguish *dosages* $$\left[ {\begin{array}{*{20}c} 1 & 0 & 0 \\ \end{array} } \right]$$ from $$\left[ {\begin{array}{*{20}c} {1/3} & {1/3} & {1/3} \\ \end{array} } \right]$$, or $$\left[ {\begin{array}{*{20}c} {f_{A}^{2} } & {2f_{A} \left( {1 - f_{A} } \right)} & {\left( {1 - f_{A} } \right)^{2} } \\ \end{array} } \right]$$ respectively, we were guided by the well-established Herfindahl–Hirschman Index (HHI) [26]. HHI is a concentration measure for distributions of discrete random variables with *k* possible realisations, defined as $= \sum\nolimits_{i = 1}^{k} {p_{k}^{2} }$. HHI ranges from 1 (if $p_{j} = 1$ and $p_{k \ne j} = 0$; alike (i)) to 1/*k* (if all $p_{k} = 1/k$; alike [ii]). Because we are interested in anti-concentration, the opposite of HHI, we first define the quantity.

$Q_{i,m} = \sum\nolimits_{g = 1}^{3} {p_{g,i,m} } \left( {1 - p_{g,i,m} } \right)$ for each marker *m* and each individual *i*. $Q_{i,m}$ takes the value 0 in case of [i]: $$\left[ {\begin{array}{*{20}c} 1 & 0 & 0 \\ \end{array} } \right]$$ and the value 2/3 in case of [ii]: $$\left[ {\begin{array}{*{20}c} {1/3} & {1/3} & {1/3} \\ \end{array} } \right]$$. To achieve an *imputation accuracy measure* (*Iam*) for each marker *m*, we then rescaled the average across all individuals $\overline{Q}_{m} = \frac{1}{N}\mathop \sum \limits_{i = 1}^{N} Q_{i,m}$ to

$$Iam_{chance,m} = 1 - \frac{{\overline{Q}_{m} }}{2/3}.$$

$Iam_{chance}$ ranges from 0 (in case of [ii]: non-informative *dosages*) to 1 (in case of [i]: fully individual genotype information).

Similarly, $\overline{Q}_{m}$ can be rescaled to represent situation [iii]: $$\left[ {\begin{array}{*{20}c} {f_{A}^{2} } & {2f_{A} \left( {1 - f_{A} } \right)} & {\left( {1 - f_{A} } \right)^{2} } \\ \end{array} } \right]$$ by the index value 0. In this case $Q_{i,m}$ takes the value

$$\begin{aligned} Q_{HWE,m} & = f_{A}^{2} \left( {1 - f_{A}^{2} } \right) + \left[ {2f_{A} \left( {1 - f_{A} } \right)\left( {1 - \left( {2f_{A} \left( {1 - f_{A} } \right)} \right)} \right)} \right] \\ & \quad + \left( {1 - f_{A} } \right)^{2} \left( {1 - \left( {1 - f_{A} } \right)^{2} } \right) \\ & = \left( { - 2f_{A} } \right)\left( {f_{A} - 1} \right)\left( {3f_{A}^{2} - 3f_{A} + 2} \right) \\ \end{aligned}$$

This alternative of the *imputation accuracy measure* (*Iam*) can be straightforwardly calculated by

$$Iam_{HWE,m} = 1 - \frac{{\overline{Q}_{m} }}{{Q_{HWE,m} }}$$

**Fig. 4:**
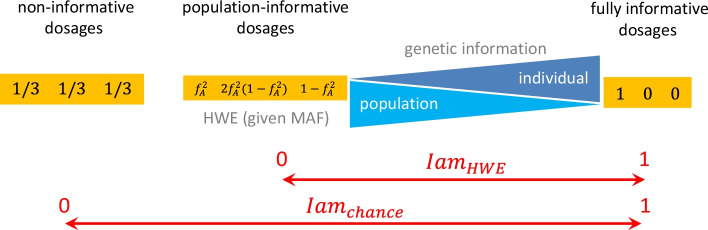
From *dosages* to *Iam*-indices. MAF/*f*_*A*_ minor allele frequency; HWE Hardy–Weinberg equilibrium; *Iam* imputation accuracy measure

Figure 4 visually presents these definitions of *Iam*.

$Iam_{chance} = 0$ indicates that the 3 genotypes are equally likely, averaged over all individuals. Therefore, imputation did not contribute any information at all.

$Iam_{HWE} = 0$ indicates that the genotypes are just as likely as under the HWE, averaged over all individuals. Therefore, imputation contributes only information of MAF in the population (respectively the reference sample), but not for further individual-specific information.

The computation of both *Iam* indices requires only the *dosages* provided by the imputation program used. For case–control or cross-sectional studies MAF can by estimated by averaging the allele doses across all individuals, using the same data:

$$\hat{f}_{A} = {\raise0.7ex\hbox{${\mathop \sum \nolimits_{i = 1}^{N} {\varvec{d}}_{{{\varvec{i}},{\varvec{m}}}} }$} \!\mathord{\left/ {\vphantom {{\mathop \sum \nolimits_{i = 1}^{N} {\varvec{d}}_{{{\varvec{i}},{\varvec{m}}}} } {2N}}}\right.\kern-\nulldelimiterspace} \!\lower0.7ex\hbox{${2N}$}}.$$

$\hat{f}_{A}$ will be calculated fair enough for the outlined purpose even for markers that are associated to a trait and therefore have different MAFs between affected and unaffected individuals. The same applies in the presence of low grade hidden relationships. However, if the study sample consists of relatives, it is advisable to consider only unrelated founders for the estimation of $\hat{f}_{A}$.

$Q_{HWE,i,m}$ depends on MAF. For rare markers $Q_{HWE,i,m}$ is much closer to 0 than for common markers (see Table 3). In case all dosages correspond to HWE (as in situation [iii]) $Iam_{chance}$ for common markers is close to 0 (indicating inaccurate imputation), whereas for rare markers it is close to 1 (misleadingly indicating accurate imputation). This shows that it is fairly hard to determine the content of individual-specific information in the triplet of dosages of rare markers.

**Table 3:** Q and *Iam*_*chance*_ by MAF

MAF	Q_chance_	Q_HWE_	*Iam*_*chance*_ based on Q_HWE_
50%	0.667	0.625	0.0625
40%	0.667	0.614	0.0784
30%	0.667	0.575	0.1369
20%	0.667	0.486	0.2704
10%	0.667	0.311	0.5329
5%	0.667	0.176	0.7353
1%	0.667	0.039	0.9415
0.1%	0.667	0.0040	0.9940
0.01%	0.667	0.0004	0.9994
0.001%	0.667	0.00004	0.9999
0.0001%	0.667	0.000004	1.0000

MAF: minor allele frequency ($f_{A}$), Q_chance_ refers to a dosage of $$\left[ {\begin{array}{*{20}c} {1/3} & {1/3} & {1/3} \\ \end{array} } \right]$$; Q_HWE_ refers to a dosage of $$\left[ {\begin{array}{*{20}c} {f_{A}^{2} } & {2f_{A}^{2} \left( {1 - f_{A} } \right)^{2} } & {1 - f_{A}^{2} } \\ \end{array} } \right]$$

It is possible that $Iam_{HWE,i,m}$ takes negative values, if the majority of triplets of *dosages* can be located between the non-informative and population-informative case. This might be caused by genotyping errors as well as by small deviations between sample and population MAF or locally increased inbreeding coefficients in the source population. Some values of $Q_{i,m}$ will then be between 2/3 and $Q_{HWE,i,m}$. Due to the upper mentioned shift of $Q_{HWE,i,m}$ by MAF, this is more likely for rare than for common markers. However, small negative values should be regarded as occurred by pure chance.

### Index of heterogeneity in quantities: hiQ

Inter-individual heterogeneity of dosages for a marker *m* is a second concern with respect to the usability of imputed genotypes. Consider the following example: Table 4 gives two markers with average dosages $$\left[ {\begin{array}{*{20}c} {0.6} & {0.3} & {0.1} \\ \end{array} } \right]$$ across 10 individuals. Marker 1 is not suitable for any data analysis, because all *dosages* are identical. The best guess for all individuals is genotype *aa*. In contrast, marker 2 consists of three different dosages, leading to different best guess genotypes for the individuals. This heterogeneity serves power for statistical testing.

**Table 4:** Inter-individual heterogeneity of dosages: example

ID	Marker 1			Marker 2		
	***aa***	***aA***	***AA***	***aa***	***aA***	***AA***
1	0.6	0.3	0.1	1	0	0
2	0.6	0.3	0.1	1	0	0
3	0.6	0.3	0.1	1	0	0
4	0.6	0.3	0.1	1	0	0
5	0.6	0.3	0.1	1	0	0
6	0.6	0.3	0.1	1	0	0
7	0.6	0.3	0.1	0	1	0
8	0.6	0.3	0.1	0	1	0
9	0.6	0.3	0.1	0	1	0
10	0.6	0.3	0.1	0	0	1
Avg	0.6	0.3	0.1	0.6	0.3	0.1

To construct an index of *heterogeneity in quantities* of *dosages* (*hiQ*) we compare “average dosages”(ad) across all individuals with “average of best guess dosages” (ab) applying the Hellinger H-distance. The H-distance quantifies the distance between two (trinomial) probability distributions, taking value H = 0 in case of coincident probability distributions and H = 1 if the probability vectors are perpendicular [27, 28]. Therefore, we defined

$$hiQ = 1 - \sqrt {1 - \mathop \sum \limits_{g = 1}^{3} \sqrt {f_{ad}^{{}} \left( g \right)f_{bg}^{{}} \left( g \right)} } .$$

In Table 4 the “average of best guess dosages” for marker 1 $$f_{bg} \left( g \right) = \left[ {\begin{array}{*{20}c} 1 & 0 & 0 \\ \end{array} } \right]$$ compared to the average dosages ($$f_{ad} \left( g \right) = \left[ {\begin{array}{*{20}c} {0.6} & {0.3} & {0.1} \\ \end{array} } \right]$$) yields an $hiQ = 1 - \sqrt {1 - \sqrt {0.6} } = 0.53$. This indicates a loss of heterogeneity between dosages and reduced power of a statistical test. For marker 2, where ${\text{f}}_{{{\text{ad}}}}^{{}} \left( {\text{g}} \right) = {\text{f}}_{{{\text{bg}}}}^{{}} \left( {\text{g}} \right)$, $hiQ$ takes on the value 1, indicating fully achievable inter-individual heterogeneity and no reduced power of a statistical test.

A SAS® macro far calculating *Iam*_*HWE*_, *Iam*_*chance*_ and *hiQ* based on the dosage-file as output of IMPUTE2 is included in the Additional file 1.

### Application to a sample of lung cancer patients and controls

We applied the novel indices to a dataset of the Integrative Analysis of Lung Cancer Etiology and Risk program of the International Lung Cancer Consortium (INTEGRAL-ILCCO) to examine the behaviour of *Iam hiQ*, to find appropriate thresholds for marker filtering and for comparison with an established accuracy measure. The sample comprises 14,803 lung cancer cases and 12,262 controls of European descent. They were genotyped on the OncoArray, which queried 517,482 SNPs. The array is designed to cover the whole genome (with GWAS backbone) and for fine mapping of susceptibility to common cancers as well as for de novo discovery, and hence is enriched with low frequent and rare variants [29]. About 50% of markers are considered as GWAS backbone. Details of the sample, the genotyping and the quality control are given elsewhere [30]. The OncoArray whole-genome data were imputed in a two-stage procedure, using SHAPEIT to derive phased genotypes and IMPUTEv2 to infer additional genotypes for genetic variants included in the 1000 Genomes Project (phase 3 panel) [6, 15]. We restricted calculations and comparisons to markers of the autosomes. A total of n = 10,427,599 SNPs were finally included in this quality assessment. Presumably difficult to impute, due to their MAF, are 5,226,623 of these SNPs (50%) with a MAF lower than 1% and 1,500,835 SNPs with a MAF between 1 and 5% (Additional file 2).

## Supplementary Information

**Additional file 1.** A macro for SAS® 9.4 to calculate the measures IamHWE, Iamchance and hiQ for autosomal markers based on the dosagefile. Finally, tables and figures are given with markers and regions of low accuracy.

**Additional file 2.** Other members (not co-authors) of the International Lung Cancer Consortium (ILCCO).

## Data Availability

The data that support the findings of this study are available from ILCCO/INTEGRAL but restrictions apply to the availability of these data, which were used under license for the current study, and so are not publicly available. Data are however available from the authors upon reasonable request and with permission of ILCCO/INTEGRAL.

## Abbreviations

Iam: Imputation accuracy measure
hiQ: Heterogeneity in quantities of dosages
ILCCO: International Lung Cancer Consortium
SNP: Single nucleotide polymorphism
SV: Structural variation
GWAS: Genome-wide association studies
MAF: Minor allele frequency
HWE: Hardy–Weinberg disequilibrium
