# Supplementary material for: Iam hiQ—a novel pair of accuracy indices for imputed genotypes
Source: BMC Bioinformatics. 2022 Jan 24;23:50. doi: 10.1186/s12859-022-04568-3 (PMC8785528; doi:10.1186/s12859-022-04568-3)
Supplement: Supplementary file 1 — Additional file 1. A macro for SAS® 9.4 to calculate the measures IamHWE, Iamchance and hiQ for autosomal markers based on the dosagefile. Finally, tables and figures are given with markers and regions of low accuracy. [file 12859_2022_4568_MOESM1_ESM.docx]

Supplement to

***Iam hiQ* – A novel pair of accuracy indices for imputed genotypes**

Albert Rosenberger et al. on behalf of the INTEGRAL-ILCCO consortium

# Usability

We investigated the usability of the proposed indices in contrast to *info* by simulation. Usability was considered in terms of discrimination between sufficient and insufficient imputation, rather than in terms of validity of imputation because validity is a characteristic of the imputation routine (e.g. IMPUTE2).

## Method

We defined eight (two times four) independent scenarios by haplotype-blocks from two common genotyped tagSNPs (alleles T and A) flanking one intermediate marker for imputation (imSNP: alleles G and C). The scenarios differ with regard to the number of haplotypes, minor allele frequencies (MAFs) and linkage disequilibrium (LD), displayed in *Usability Table 1* and *2*, and *Usability Figure 1*. All markers were simulated in Hardy-Weinberg equilibrium. Two scenarios each form a pair (a scene), consisting of a scenario in which the missing marker can be imputed sufficiently / better and one scenario in which the missing marker can be imputed insufficiently / worse. For each scenario we simulated 100 variant imputations and assessed their quality calculating ${Iam}_{HWE}$, ${Iam}_{chance}$, $hiQ$ and *info.* Thus, for each scene, 200 values of each accuracy index are available to differentiate between 100 “sufficient” and 100 “insufficient” scenarios. To visualize the ability to discriminate a sufficient from an insufficient scenario (usability) we plotted comparative receiver operation curves (one ROC per index) for each scene.

Simulation details: We first simulated an artificial reference population of 100,000 individuals for all 3x8=14 genotypes and built a random reference sample of 1000 individuals. We than drew k=100 random samples of size n=500 individuals comprising the genotypes of 2x8=16 tagSNPs each. This genotype data set was then phased using SHAPEIT and missing genotypes were imputed with IMPUTE2. For all 8x100 imputed markers we calculated ${Iam}_{HWE}$, ${Iam}_{chance}$ and $hiQ$, and extracted *info* from the IMPUTE2 output files.

Usability Table 1: Definition of scenes and scenarios

**Scenes 1 and 2**: common tagSNPs (MAF: 0.45 – 0.50) – common imSNP (MAF: 0.45 – 0.50)

Scene 1: 4 haplotypes (2 predominant haplotypes) – tagSNPs in strong LD to each other;

Scenario 1.1 (worse): weaker LD between both tagSNPs and imSNP*
Scenario 1.2 (better): stronger LD between both tagSNPs and imSNP*

Scene 2: 8 haplotypes (no predominant haplotype) – tagSNPs in weak or no LD to each other;

Scenario 2.1 (worse): weaker LD between of one tagSNP and imSNP*
Scenario 2.2 (better): stronger LD between both tagSNPs and imSNP*

**Scenes 3 and 4**: common tagSNPs (MAF: 0.45 – 0.50) – less frequent imSNP (MAF: 0.02 – 0.04)

Scene 3: 4 haplotypes (2 predominant haplotypes) – tagSNPs in strong LD to each other;

Scenario 3.1 (worse): weaker LD between both tagSNPs and imSNP*
Scenario 3.2 (better): stronger LD between one tagSNPs and imSNP*

Scene 4: 8 haplotypes (no predominant haplotype) – tagSNPs in no or weak LD to each other;

Scenario 4.1 (worse): weaker LD between of only tagSNP and imSNP*
Scenario 4.2 (better): stronger LD between both tagSNPs and imSNP*

* imSNP: marker for imputation

Usability Table 2: Definition of scenes and scenarios

|  | scene 1 | | | scene 2 | | scene 3 | | | scene 4 | |
| --- | --- | --- | --- | --- | --- | --- | --- | --- | --- | --- |
|  | 1.1. worse | 1.2. better | 2.1. worse | | 2.2. better | 3.1. worse | 3.2. better | 4.1. worse | | 4.2 better |

**Haplotypes with simulated frequency**

| ACA | 45.0% | 47.5% | 15.0% | 13.6% | 1.5% | 2.0% | <1% | <1% |
| --- | --- | --- | --- | --- | --- | --- | --- | --- |
| ACT | -- | -- | 15.0% | 13.6% | -- | 2.0% | <1% | 2.9% |
| AGA | 5.0% | 2.5% | 10.0% | 9.1% | 48.5% | 40.8% | 24.5% | <1% |
| AGT | -- | -- | 10.0% | 13.6% | -- | 6.1% | 24.5% | 38.5% |
| TCA | -- | -- | 10.0% | 13.6% | -- | -- | <1% | <1% |
| TCT | 5.0% | 2.5% | 10.0% | 9.1% | 1.5% | -- | <1% | <1% |
| TGA | -- | -- | 15.0% | 13.6% | -- | 8.2% | 24.5% | 38.5% |
| TGT | 45.0% | 47.5% | 15.0% | 13.6% | 48.5% | 40.8% | 24.5% | 19.3% |

**Minor allele frequency in reference panel**

| tagSNP | 0.495 | 0.483 | 0.496 | 0.493 | 0.498 | 0.498 | 0.466 | 0.428 |
| --- | --- | --- | --- | --- | --- | --- | --- | --- |
| *imSNP** | 0.498 | 0.487 | 0.470 | 0.491 | 0.037 | 0.038 | 0.022 | 0.036 |
| tagSNP | 0.495 | 0.483 | 0.489 | 0.497 | 0.498 | 0.494 | 0.457 | 0.372 |

* imSNP: marker for imputation

Usability Figure 1: LD-Pattern in reference panel

| scene 1 | | scene 2 | | scene 3 | | scene 4 | |
| --- | --- | --- | --- | --- | --- | --- | --- |
| 1.1. worse | 1.2. better | 2.1. worse | 2.2. better | 3.1. worse | 3.2. better | 4.1. worse | 4.2 better |

**
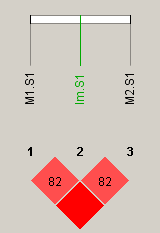

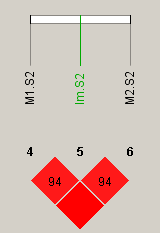

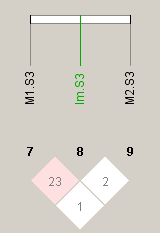

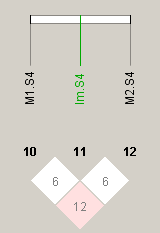

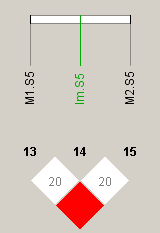

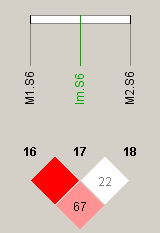

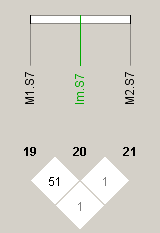

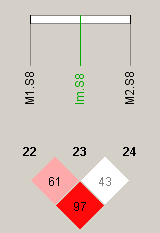
**

LD is presented as D’

## Result

*Info* correlates in this artificial data set, across all four scenes, strongest to ${I'am}_{HWE}$ (r=0.97), followed by $hiQ$ (r=0.90) and ${I'am}_{chance}$ (r=0.80). This is similar to the real data example presented in the main text. The correlation of ${I'am}_{HWE}$ to $hiQ$ and to ${I'am}_{chance}$ was in-between (0.92 and 0.82 respectively).

In all four scenes the ROC curves of ${I'am}_{HWE}$ and ${I'am}_{chance}$ are identical, hereinafter referred together as $I'am$. Scene >1< can be regarded as proof of general functionality for common SNPs, since all but one indices truly indicate almost perfect discrimination between sufficient and insufficient imputation (all AUC=1, see *Usability Figure 2* and *Usability Table 3*) in the presence of predominant haplotypes. Solely *hiQ* shows a lower discriminability relative to the other accuracy scores (AUC=0.896). In scene >2< $I'am$ and *info* are almost equally sufficient (AUC~0.82), while *hiQ* shows again a lower discriminability relative to the other accuracy scores (AUC=0.748).

In contrast, *hiQ* shows a uniformly higher level of discriminability than *info* and *Iam* in scene >3<, where the MAF of the SNP to be imputed is low but two haplotypes predominate. *Info* and *Iam* remain comparable, given the pair of scenarios was set very close. In the more complex scene >4<, *info* and *hiQ* seems to be comparable but consistently show a slightly higher degree of distinctness than *Iam*.

Usability Figure 2: ROC to visualize discrimination between sufficient and insufficient imputation

| scene 1 | scene 2 | scene 3 | scene 4 |
| --- | --- | --- | --- |


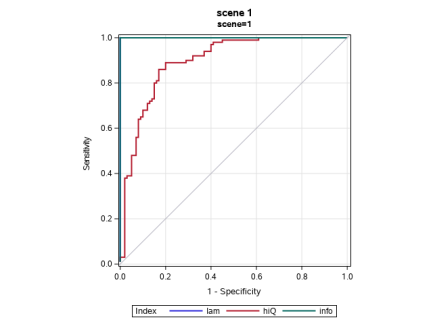

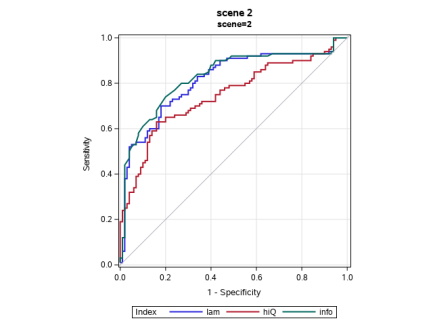

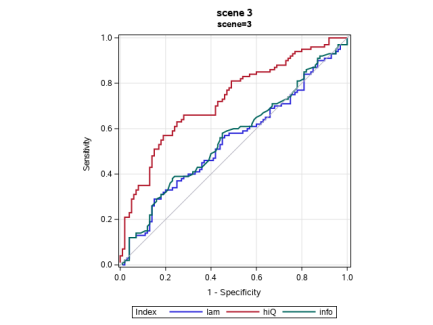

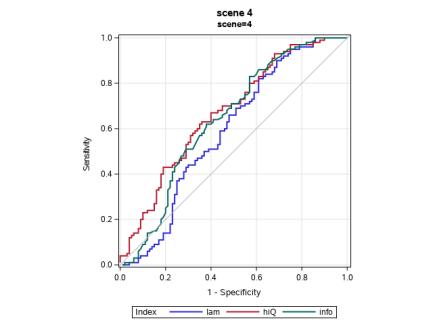


Usability Table 3: AUC-ROC to quantify discrimination between sufficient and insufficient imputation

| Scene | Score | AUC-ROC | Standard Error | 95% Wald Confidence Limits | |
| --- | --- | --- | --- | --- | --- |
| 1 | ${I'am}_{HWE}$ | **1** | -- | -- | -- |
|  | ${I'am}_{chance}$ | **1** | -- | -- | -- |
|  | $hiQ$ | 0.8960 | 0.0226 | 0.8516 | 0.9404 |
|  | *info* | **1** | -- | -- | -- |
| 2 | ${I'am}_{HWE}$ | 0.8139 | 0.0313 | 0.7526 | 0.8752 |
|  | ${I'am}_{chance}$ | 0.8139 | 0.0313 | 0.7526 | 0.8752 |
|  | $hiQ$ | 0.7478 | 0.0351 | 0.6789 | 0.8167 |
|  | *info* | **0.8293** | 0.0304 | 0.7698 | 0.8888 |
| 3 | ${I'am}_{HWE}$ | 0.5418 | 0.0411 | 0.4613 | 0.6223 |
|  | ${I'am}_{chance}$ | 0.5418 | 0.0411 | 0.4613 | 0.6223 |
|  | $hiQ$ | **0.7250** | 0.0357 | 0.6549 | 0.7951 |
|  | *info* | 0.5565 | 0.0409 | 0.4763 | 0.6367 |
| 4 | ${I'am}_{HWE}$ | 0.5901 | 0.0410 | 0.5097 | 0.6705 |
|  | ${I'am}_{chance}$ | 0.5901 | 0.0410 | 0.5097 | 0.6705 |
|  | $hiQ$ | **0.6699** | 0.0380 | 0.5954 | 0.7444 |
|  | *info* | 0.6397 | 0.0397 | 0.5619 | 0.7174 |

Bold numbers display the largest AUC-ROC (area under the receiver operating characteristic curve) within a scene.

## Conclusion

*Info* and *Iam* appear to be comparable in terms of the distinction between sufficient and insufficient imputation for common SNPs. However, *hiQ* seems to be superior if the minor allele frequency (MAF) of the imputed marker is low.

Nevertheless, please note: Simulation studies are computer experiments with data generated from known probability distributions by means of pseudo-random samples. The performance of statistical methods is only examined in specific predefined scenarios. Therefore, the results of simulations are limited to the scenarios selected for data generation. Simulations give different results when based on different random numbers generating processes, and conclusions from them are therefore always limited and subject to uncertainty (1).

## Reference

1. Morris TP, White IR, Crowther MJ. Using simulation studies to evaluate statistical methods. Statistics in Medicine. 2019;38(11):2074–102.

# SAS-MACRO

The following macro for SAS^®^ 9.4 calculates the measures *Iam_HWE_*, *Iam_chance_* and *hiQ* for autosomal markers based on the *dosage-file* as output of IMPUTE2. To run the macro one needs to specify:

dose_path: the physical path of the dosage-file

dose_file: the physical name of the dosage-file

out_file: the physical name of the output-file, that is stored in the dose_path

**%macro** IamHIQ(dose_path,dose_file,out_file);

****** determine number of individuals in dosage file *********;

****************************************************************; option;

proc printto log=sinnlos; run;

data _null_;

length lines **8** cmd $**300**;

cmd = catx(' ','head -n 1 ',"&dose_path.&dose_file..imputed",' | wc -w');

infile cmd pipe filevar=cmd truncover;

input lines @;

if _N_=**1** then do;

anz_iid=(lines-**5**)/**3**;

call symputx('anz_iid',anz_iid);

call symputx('firstvar',**6**);

call symputx('lastvar',**5**+**3***anz_iid);

end;

run;

proc printto log=log; run;

%put anz_iid: &anz_iid. &firstvar. - &lastvar.;

****** read dosage file --> calculate Iam hiQ ***********;

****** export as xx.IamHiQ and as SAS-file ************;

***************************************************************; option;

libname dosage "&dose_path.";

options;

data dosage.&out_file._IamHiQ(keep=snp_id rs_id position a0 a1 MAF Iam_chance Iam_HWE hiQ);

%let _EFIERR_ = 0; /* set the ERROR detection macro variable */

infile "&dose_path.&dose_file..imputed"

delimiter = ' ' MISSOVER DSD lrecl=**32767** firstobs=**1** ;/*obs=100*/;

informat snp_id $3. rs_id $45. position best32. a0 a1 $1.

MAF Q Q_HWE 8.6

Hsum Iam_chance Iam_HWE hiQ 5.3

VAR&firstvar.-VAR&lastvar. best32. ;

format n comma9.0 MAF percent.

snp_id $3. rs_id $15. position best. a0 a1 $1. VAR&firstvar.-VAR&lastvar. 6.4

bd1-bd3 ad1-ad3 Q Q_HWE Hsum Iam_chance Iam_HWE hiQ best6.;

input snp_id $ rs_id $ position a0 $ a1 $ VAR&firstvar.-VAR&lastvar. ;

if strip(rs_id)='' then delete;

array dd {&anz_iid.,**3**} VAR&firstvar.-VAR&lastvar. ;

array bd {**3**] bd1-bd3 (**0** **0** **0**);

array ad {**3**] ad1-ad3 (**0** **0** **0**);

MAF=**0**;

do i=**1** to &anz_iid.;

MAF=sum(MAF,**0***dd[i,**1**],**1***dd[i,**2**],**2***dd[i,**3**]);

end;

MAF=MAF/**2**/&anz_iid.;

If MAF=**0** then MAF=**0.5**/**2**/&anz_iid.; *** correction for monomorphic markers ****;

If MAF=**1** then MAF=**1**-**0.5**/**2**/&anz_iid.; *** correction for monomorphic markers ****;

Q=**0**;

do i=**1** to &anz_iid.;

*** calculate best guess dosage ***;

b1=**0**; b2=**0**; b3=**0**;

if dd[i,**1**]=max(dd[i,**1**],dd[i,**2**],dd[i,**3**]) then b1=**1**;

if dd[i,**2**]=max(dd[i,**1**],dd[i,**2**],dd[i,**3**]) then b2=**1**;

if dd[i,**3**]=max(dd[i,**1**],dd[i,**2**],dd[i,**3**]) then b3=**1**;

b_sum=sum(b1,b2,b3);

b1=b1/b_sum;

b2=b2/b_sum;

b3=b3/b_sum;

*** sum of best guess dosage ***;

bd[**1**]=sum(bd[**1**],b1);

bd[**2**]=sum(bd[**2**],b2);

bd[**3**]=sum(bd[**3**],b3);

*** sum of dosage ***;

ad[**1**]=sum(ad[**1**],dd[i,**1**]);

ad[**2**]=sum(ad[**2**],dd[i,**2**]);

ad[**3**]=sum(ad[**3**],dd[i,**3**]);

*** calculate Q ***;

Q=sum(Q,dd[i,**1**]*(**1**-dd[i,**1**]),

dd[i,**2**]*(**1**-dd[i,**2**]),

dd[i,**3**]*(**1**-dd[i,**3**]));

end;

**** calculate means from sums ****;

Q=Q/&anz_iid.;

bdsum=sum(of bd[*]); *** correction of round-off errors / denominator instead of &anz_iid. ****;

bd[**1**]=bd[**1**]/bdsum;

bd[**2**]=bd[**2**]/bdsum;

bd[**3**]=bd[**3**]/bdsum;

adsum=sum(of ad[*]); *** correction of round-off errors / denominator instead of &anz_iid. ****;

ad[**1**]=ad[**1**]/adsum;

ad[**2**]=ad[**2**]/adsum;

ad[**3**]=ad[**3**]/adsum;

Q_HWE=**2***MAF*(**1**-MAF)*(**3***MAF****2**-**3***MAF+**2**);

Iam_chance=**1**-Q/(**2**/**3**);

if Q_HWE>**0** then Iam_HWE=**1**-Q/Q_HWE;

Hsum=min(**1**,sum(sqrt(ad[**1**]*bd[**1**]),sqrt(ad[**2**]*bd[**2**]),sqrt(ad[**3**]*bd[**3**])));

hiQ=**1**-sqrt(**1**-Hsum);

***** Export as txt-file*****;

file "&dose_path.&dose_file..IamhiQ";

if _N_=**1** then put 'snp_id rs_id position a0 a1 MAF Iam_chance Iam_HWE hiQ';

put snp_id rs_id position a0 a1 MAF Iam_chance Iam_HWE hiQ;

run;

****** print calculate Iam hiQ *************;

****************************************************************; option;

proc print data=dosage.&out_file._IamHiQ noobs;

var snp_id rs_id position a0 a1 MAF Iam_chance Iam_HWE hiQ

run;

**%mend** IamHIQ;

# Comparison of *Iam* and *hiQ*

Additional file 1: Table S1 *Iam_HWE_* contrasted with *hiQ*

|  | ***Iam_HWE_*** | | | | | | | | | | | | | | | |  |
| --- | --- | --- | --- | --- | --- | --- | --- | --- | --- | --- | --- | --- | --- | --- | --- | --- | --- |
|  | **negative** | **0.0-<0.1** | **0.1-<0.2** | **0.2-<0.25** | **0.25-<0.3** | **0.3-<0.4** | **0.4-<0.5** | **0.5-<0.6** | **0.6-<0.7** | **0.7-<0.75** | **0.75-<0.8** | **0.8-<0.85** | **0.85-<0.9** | **0.9-<0.95** | **0.95-<1** | **1*** | **All** |
| ***hiQ*** | **N**  **cum-%**  **%-info** | **N**  **cum-%**  **%-info** | **N**  **cum-%**  **%-info** | **N**  **cum-%**  **%-info** | **N**  **cum-%**  **%-info** | **N**  **cum-%**  **%-info** | **N**  **cum-%**  **%-info** | **N**  **cum-%**  **%-info** | **N**  **cum-%**  **%-info** | **N**  **cum-%**  **%-info** | **N**  **cum-%**  **%-info** | **N**  **cum-%**  **%-info** | **N**  **cum-%**  **%-info** | **N**  **cum-%**  **%-info** | **N**  **cum-%**  **%-info** | **N**  **cum-%**  **%-info** | **N** |
| **0.4-<0.5** |  | 784  <0.1%  0% |  |  |  |  |  |  |  |  |  |  |  |  |  |  | **784** |
|  |  |  |  |  |  |  |  |  |  |  |  |  |  |  |  |  |  |
| **0.5-<0.6** |  | 700  <0.1%  0% |  |  |  |  |  |  |  |  |  |  |  |  |  |  | **700** |
| **0.6-<0.7** |  | 1,000  <0.1%  0% | 19  <0.1%  0% |  |  |  |  |  |  |  |  |  |  |  |  |  | **1,019** |
| **0.7-<0.75** |  | 801  <0.1%  0% | 54  <0.1%  0% |  |  |  |  |  |  |  |  |  |  |  |  |  | **855** |
| **0.75-<0.8** |  | 861  <0.1%  0% | 94  <0.1%  0% | 3  <0.1%  0% |  |  |  |  |  |  |  |  |  |  |  |  | **958** |
| **0.8-<0.85** | 272  <0.1%  0% | 643  <0.1%  0% | 417  0.1%  0% | 45  0.1%  0% | 18  0.1%  0% | 4  0.1%  0% |  |  |  |  |  |  |  |  |  |  | **1,399** |
| **0.85-<0.9** | 903  <0.1%  0% | 866  0.1%  0% | 406  0.1%  0% | 653  0.1%  0% | 956  0.1%  0% | 994  0.1%  0% | 49  0.1%  0% | 2  0.1%  0% |  |  |  |  |  |  |  |  | **4,829** |
| **0.9-<0.95** | 1,972  <0.1%  0% | 2,118  0.1%  0% | 627  0.1%  0% | 300  0.1%  0% | 1,644  0.2%  0% | 10,332  0.3%  0% | 12,018  043%  0% | 3,171  0.4%  0% | 273  0.4%  0% | 16  0.4%  <0.1% | 7  0.4%  <0.1% |  |  |  |  |  | **32,478** |
| **0.95-<1.0** | 1,761  <0.1%  0% | 13,779  0.3%  0% | 33,698  0.6%  0% | 48,171  1.1%  0% | 92,235  2.0%  0% | 403,504  6.0%  0% | 679,310  12.6%  <0.1% | 727,860  19.6%  <0.1% | 704,259  26.3%  <0.1% | 334,333  29.6%  0.2% | 303,985  32.5%  1.3% | 249,988  34.9%  5.0% | 194,721  36,7%  10.0% | 191,258  38.6%  17.2% | 349,192  41.9%  43.7% | 130,112  43.2%  45.7% | **4,458,166** |
| **1.0*** |  | 41 0.3% 0% | 2,681 0.6% 0% | 5,125 1.1% 0% | 10,575 2.2% 0% | 71,753 6.8% 0% | 209,348  15.5%  <0.1% | 304,154  25.4%  <0.1% | 328,620  35.3%  <0.1% | 189,950  40.3%  0.2% | 238,318  45.5%  1.3% | 323,744  51.0%  5.3% | 452,150  57.2%  10.7% | 724,635  66.0%  18.7% | 2,143,291  89.9%  48.6% | 922,026  100%  50.9% | **5,926,411** |
| **All** | **4,908** | **21,593** | **37,996** | **54,297** | **105,428** | **486,587** | **900,725** | **1,035,187** | **1,033,152** | **524,299** | **542,310** | **573,732** | **646,871** | **915,893** | **2,492,483** | **1,052,138** | **10,427,599** |

**cum-%:** : cumulated proportion of all markers belonging to cells to the left und above the indicating cell;
**%-info**: proportion of marker belonging to the indicated cell with *info*≥0.8.; * >0.995

# Markers and regions of low accuracy

Additional file 1: Table S2 *Iam* sections

|  |  | cold | | | hot | | | very hot | | |
| --- | --- | --- | --- | --- | --- | --- | --- | --- | --- | --- |
| chromosome | N  sections | N  sections | N markers | | N  sections | N markers | | N  sections | N markers | |
|  |  |  | min | max |  | min | max |  | min | max |
| **All** | 9,201 | 4,427 | 1 | 259,790 | 4,603 | 1 | 3,218 | 171 | 1 | 6,030 |
| **1** | 775 | 381 | 1 | 259,790 | 388 | 1 | 2,313 | 6 | 1 | 3,772 |
| **2** | 470 | 233 | 1 | 157,307 | 235 | 1 | 966 | 2 | 3 | 3,545 |
| **3** | 180 | 90 | 1 | 146,181 | 90 | 1 | 136 |  |  |  |
| **4** | 374 | 187 | 1 | 128,016 | 187 | 1 | 1,167 |  |  |  |
| **5** | 183 | 91 | 1 | 144,454 | 92 | 1 | 243 |  |  |  |
| **6** | 164 | 82 | 1 | 178,987 | 82 | 1 | 164 |  |  |  |
| **7** | 720 | 360 | 1 | 104,708 | 360 | 1 | 126 |  |  |  |
| **8** | 398 | 199 | 1 | 83,900 | 199 | 1 | 834 |  |  |  |
| **9** | 437 | 142 | 1 | 124,506 | 219 | 1 | 148 | 76 | 1 | 6,030 |
| **10** | 294 | 147 | 1 | 182,491 | 147 | 1 | 890 |  |  |  |
| **11** | 1,283 | 632 | 1 | 162,101 | 641 | 1 | 241 | 10 | 1 | 4 |
| **12** | 149 | 74 | 1 | 151,950 | 75 | 1 | 238 |  |  |  |
| **13** | 214 | 107 | 1 | 158,509 | 107 | 1 | 121 |  |  |  |
| **14** | 298 | 149 | 1 | 174,951 | 149 | 1 | 3,218 |  |  |  |
| **15** | 244 | 118 | 1 | 79,309 | 122 | 1 | 2,014 | 4 | 2 | 2,642 |
| **16** | 1,343 | 598 | 1 | 61,727 | 672 | 1 | 252 | 73 | 1 | 75 |
| **17** | 357 | 178 | 1 | 63,321 | 179 | 1 | 245 |  |  |  |
| **18** | 275 | 138 | 1 | 137,118 | 137 | 1 | 59 |  |  |  |
| **19** | 447 | 223 | 1 | 32,005 | 224 | 1 | 175 |  |  |  |
| **20** | 78 | 39 | 1 | 41,291 | 39 | 1 | 34 |  |  |  |
| **21** | 234 | 117 | 1 | 48,667 | 117 | 1 | 896 |  |  |  |
| **22** | 284 | 142 | 1 | 70,118 | 142 | 1 | 1,674 |  |  |  |

*Iam_HWE_*: cold *Iam_HWE_*(ewma)> 0.47 hot *Iam_HWE_*(ewma)<= 0.47 very hot *Iam_HWE_*(ewma)<= 0.23

Additional file 1: Table S3 *hiQ* sections

|  |  | cold | | | hot | | | very hot | | |
| --- | --- | --- | --- | --- | --- | --- | --- | --- | --- | --- |
| chromosome | N  sections | N  sections | N markers | | N  sections | N markers | | N  sections | N markers | |
|  |  |  | min | max |  | min | max |  | min | max |
| **All** | 5,801 | 2,902 | 1 | 493,993 | 2,899 | 1 | 6,181 |  |  |  |
| **1** | 815 | 407 | 1 | 215,969 | 408 | 1 | 4,376 |  |  |  |
| **2** | 364 | 182 | 1 | 354,867 | 182 | 1 | 3,557 |  |  |  |
| **3** | 111 | 56 | 1 | 416,639 | 55 | 1 | 105 |  |  |  |
| **4** | 324 | 162 | 1 | 493,993 | 162 | 1 | 390 |  |  |  |
| **5** | 96 | 48 | 1 | 289,128 | 48 | 1 | 50 |  |  |  |
| **6** | 174 | 87 | 1 | 178,990 | 87 | 1 | 75 |  |  |  |
| **7** | 273 | 137 | 1 | 143,501 | 136 | 1 | 76 |  |  |  |
| **8** | 256 | 128 | 1 | 268,957 | 128 | 1 | 212 |  |  |  |
| **9** | 113 | 56 | 1 | 251,107 | 57 | 1 | 6,181 |  |  |  |
| **10** | 418 | 209 | 1 | 252,751 | 209 | 1 | 96 |  |  |  |
| **11** | 397 | 199 | 1 | 304,344 | 198 | 1 | 189 |  |  |  |
| **12** | 116 | 58 | 1 | 316,860 | 58 | 1 | 74 |  |  |  |
| **13** | 170 | 85 | 1 | 315,765 | 85 | 1 | 38 |  |  |  |
| **14** | 484 | 242 | 1 | 260,266 | 242 | 1 | 353 |  |  |  |
| **15** | 241 | 120 | 1 | 129,380 | 121 | 1 | 2,644 |  |  |  |
| **16** | 326 | 163 | 1 | 84,886 | 163 | 1 | 165 |  |  |  |
| **17** | 156 | 78 | 1 | 82,837 | 78 | 1 | 99 |  |  |  |
| **18** | 51 | 26 | 1 | 211,630 | 25 | 1 | 27 |  |  |  |
| **19** | 219 | 109 | 1 | 78,534 | 110 | 1 | 31 |  |  |  |
| **20** | 51 | 26 | 1 | 80,031 | 25 | 1 | 11 |  |  |  |
| **21** | 293 | 147 | 1 | 126,529 | 146 | 1 | 153 |  |  |  |
| **22** | 353 | 177 | 1 | 87,799 | 176 | 1 | 455 |  |  |  |

*hiQ*: cold *hiQ* (ewma)> 0.97 hot *hiQ* (ewma)<= 0.97 very hot *hiQ* (ewma)<= 0.48

Additional file 1: Table S4 Size of Sections with elevated *Iam* or *hiQ*

| N markers | *Iam* sections | | | | | | *hiQ* sections | | | |
| --- | --- | --- | --- | --- | --- | --- | --- | --- | --- | --- |
|  | cold | | hot | | very hot | | cold | | hot | |
|  | N | % | N | % | N | % | N | % | N | % |
| **1** | 1,184 | 26.7% | 1,626 | 35.3% | 47 | 27.4% | 799 | 27.5% | 968 | 33.3% |
| **2** | 449 | 10.1% | 670 | 14.5% | 21 | 12.2% |  |  |  |  |
| **3-5** | 715 | 16.1% | 878 | 19.0% | 45 | 26.3% | 367 | 12.6% | 395 | 13.6% |
| **6-20** | 808 | 18.2% | 940 | 20.4% | 23 | 13.4% | 480 | 16.5% | 553 | 19.0% |
| **>21** | 1,271 | 28.7% | 489 | 10.6% | 35 | 20.4% | 556 | 19.1% | 634 | 21.8% |

Additional file 1: Table S5 Missingness of accuracy measures per chromosome

| chromosome |  | *Iam_HWE_* | % | *hiQ* | % | *info* | % | *certainty* | % |
| --- | --- | --- | --- | --- | --- | --- | --- | --- | --- |
| all | n | 10,427,599 | -- | 10,427,599 | -- | 8,556,447 |  | 10,367,376 |  |
|  | missing | 0 |  | 0 |  | 1,871,152 | 17.90% | 60,223 | 0.60% |
| 1 | n | 827,075 |  | 827,075 |  | 673,664 |  | 886,729 |  |
|  | missing | 0 | -- | 0 | -- | 153,431 | 18.60% | 4,714 | 0.60% |
| 2 | n | 886,729 |  | 886,729 |  | 725,693 |  | 881,752 |  |
|  | missing | 0 | -- | 0 | -- | 161,036 | 18.20% | 4,977 | 0.60% |
| 3 | n | 741,115 |  | 741,115 |  | 613,539 |  | 736,768 |  |
|  | missing | 0 | -- | 0 | -- | 127,576 | 17.20% | 4,347 | 0.60% |
| 4 | n | 744,039 |  | 744,039 |  | 615,826 |  | 739,435 |  |
|  | missing | 0 | -- | 0 | -- | 128,213 | 17.20% | 4,604 | 0.60% |
| 5 | n | 662,493 |  | 662,493 |  | 546,907 |  | 658,654 |  |
|  | missing | 0 | -- | 0 | -- | 115,586 | 17.40% | 3,839 | 0.60% |
| 6 | n | 680,135 |  | 680,135 |  | 562,867 |  | 675,715 |  |
|  | missing | 0 | -- | 0 | -- | 117,268 | 17.20% | 4,420 | 0.60% |
| 7 | n | 608,856 |  | 608,856 |  | 498,045 |  | 605,374 |  |
|  | missing | 0 | -- | 0 | -- | 110,811 | 18.20% | 3,482 | 0.60% |
| 8 | n | 572,378 |  | 572,378 |  | 471,242 |  | 569,105 |  |
|  | missing | 0 | -- | 0 | -- | 101,136 | 17.70% | 3,273 | 0.60% |
| 9 | n | 458,056 |  | 458,056 |  | 373,531 |  | 455,436 |  |
|  | missing | 0 | -- | 0 | -- | 84,525 | 18.50% | 2,620 | 0.60% |
| 10 | n | 524,418 |  | 524,418 |  | 431,775 |  | 521,238 |  |
|  | missing | 0 | -- | 0 | -- | 92,643 | 17.70% | 3,180 | 0.60% |
| 11 | n | 512,601 |  | 512,601 |  | 422,525 |  | 509,537 |  |
|  | missing | 0 | -- | 0 | -- | 90,076 | 17.60% | 3,064 | 0.60% |
| 12 | n | 498,183 | -- | 498,183 | -- | 409,701 | 17.80% | 495,064 | 0.60% |
|  | missing | 0 |  | 0 |  | 88,482 |  | 3,119 |  |
| 13 | n | 373,913 |  | 373,913 |  | 311,154 |  | 371,663 |  |
|  | missing | 0 | -- | 0 | -- | 62,759 | 16.80% | 2,250 | 0.60% |
| 14 | n | 342,312 |  | 342,312 |  | 282,327 |  | 340,241 |  |
|  | missing | 0 | -- | 0 | -- | 59,985 | 17.50% | 2,071 | 0.60% |
| 15 | n | 307,558 |  | 307,558 |  | 251,973 |  | 307,558 |  |
|  | missing | 0 | -- | 0 | -- | 55,585 | 18.10% | 0 | -- |
| 16 | n | 338,758 |  | 338,758 |  | 270,652 |  | 336,666 |  |
|  | missing | 0 | -- | 0 | -- | 68,106 | 20.10% | 2,092 | 0.60% |
| 17 | n | 294,636 |  | 294,636 |  | 235,253 |  | 292,927 |  |
|  | missing | 0 | -- | 0 | -- | 59,383 | 20.20% | 1,709 | 0.60% |
| 18 | n | 292,314 |  | 292,314 |  | 241,881 |  | 290,595 |  |
|  | missing | 0 | -- | 0 | -- | 50,433 | 17.30% | 1,719 | 0.60% |
| 19 | n | 244,372 |  | 244,372 |  | 194,432 |  | 242,809 |  |
|  | missing | 0 | -- | 0 | -- | 49,940 | 20.40% | 1,563 | 0.60% |
| 20 | n | 230,100 |  | 230,100 |  | 189,574 |  | 228,803 |  |
|  | missing | 0 | -- | 0 | -- | 40,526 | 17.60% | 1,297 | 0.60% |
| 21 | n | 140,984 |  | 140,984 |  | 116,352 |  | 140,056 |  |
|  | missing | 0 | -- | 0 | -- | 24,632 | 17.50% | 928 | 0.70% |
| 22 | n | 146,574 |  | 146,574 |  | 117,554 |  | 145,646 |  |
|  | missing | 0 | -- | 0 | -- | 29,020 | 19.80% | 928 | 0.60% |

Additional file: Figure S1 Manhattan-like-plot: *Iam hiQ* by chromosomes


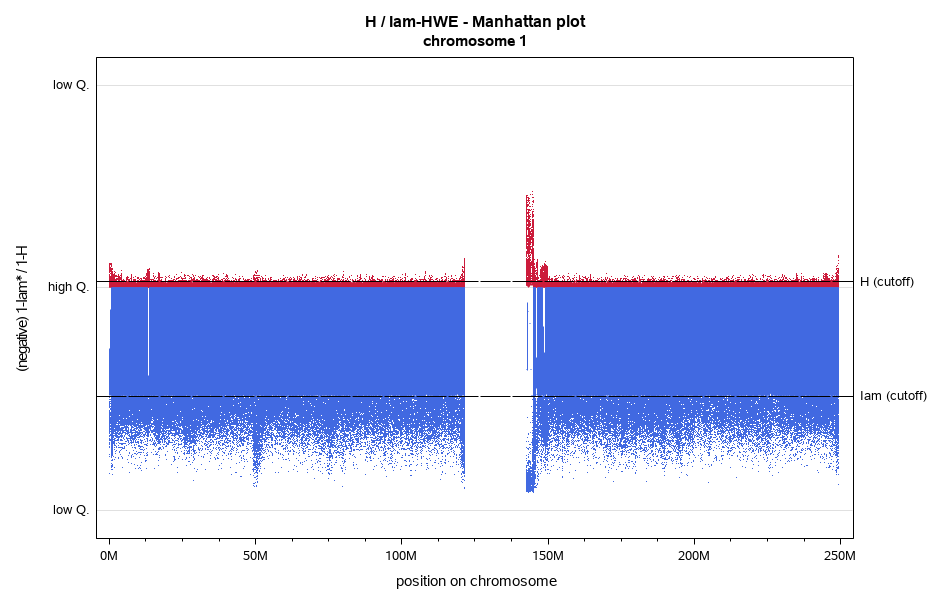

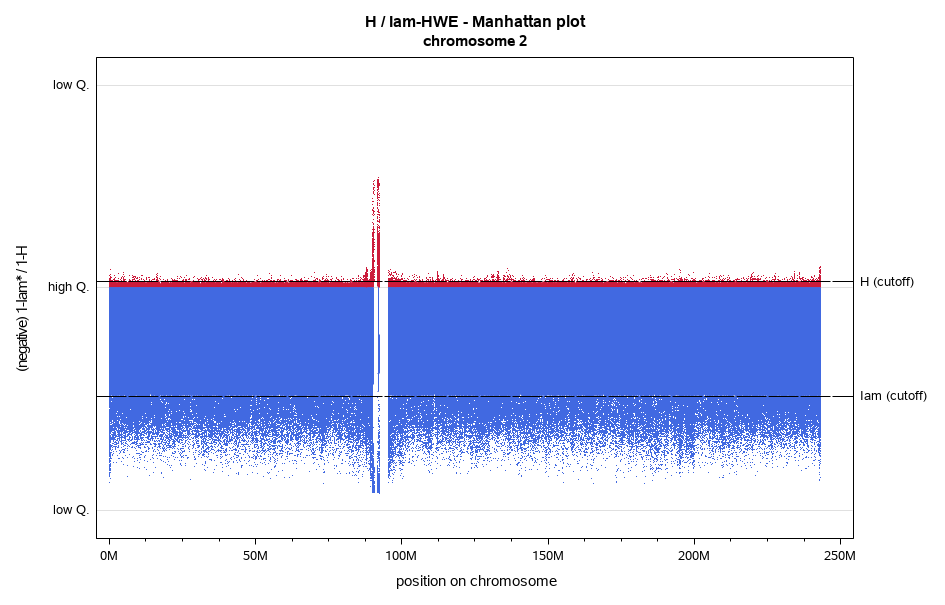

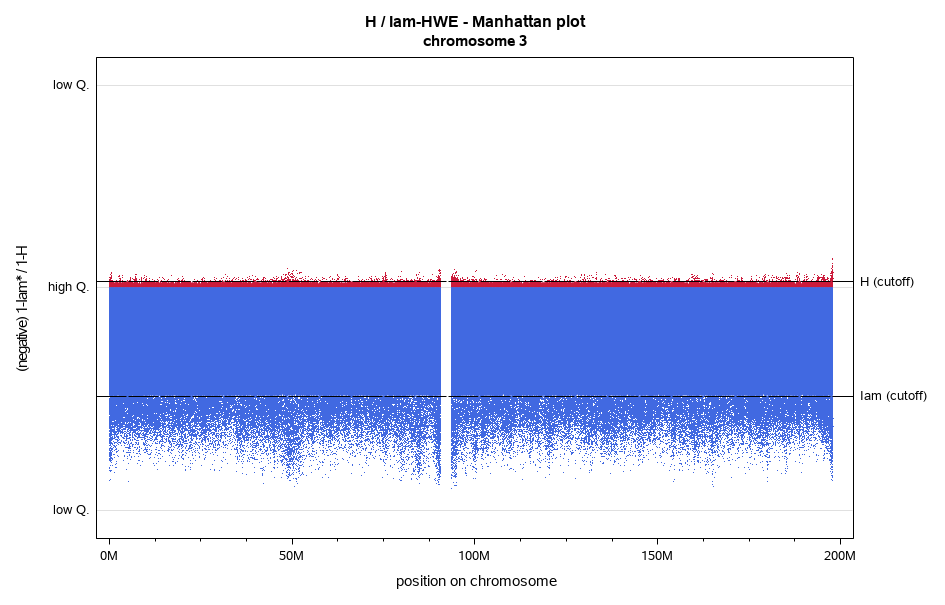

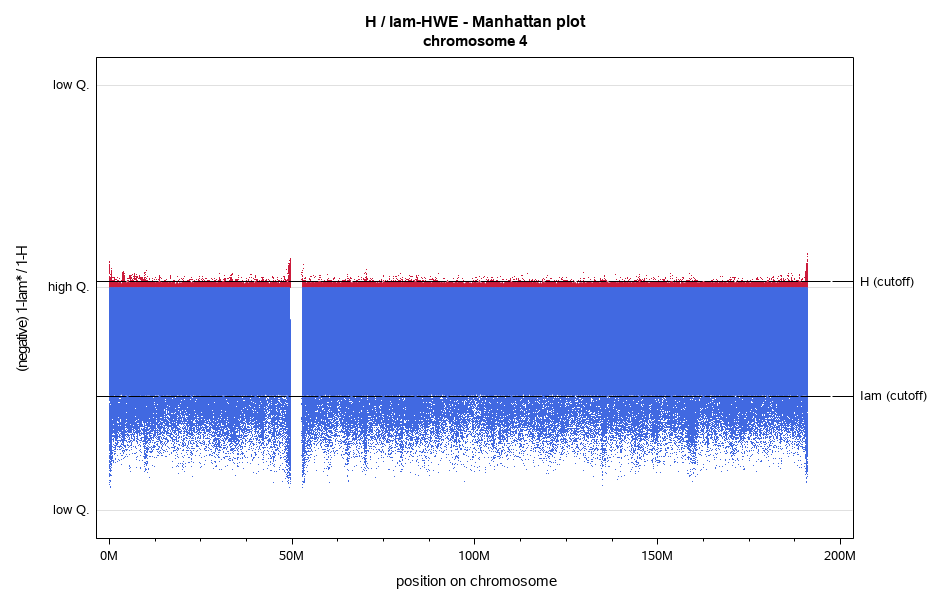

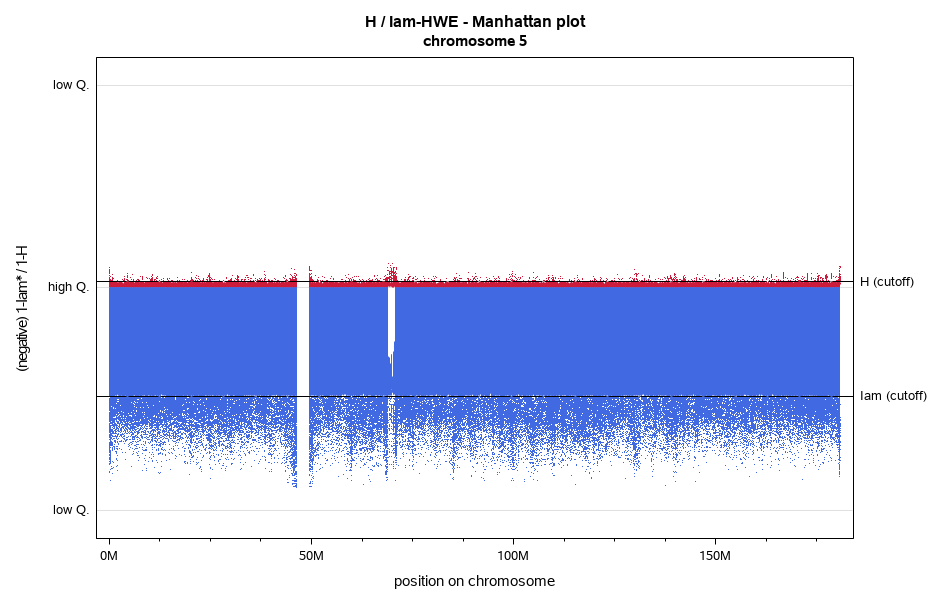

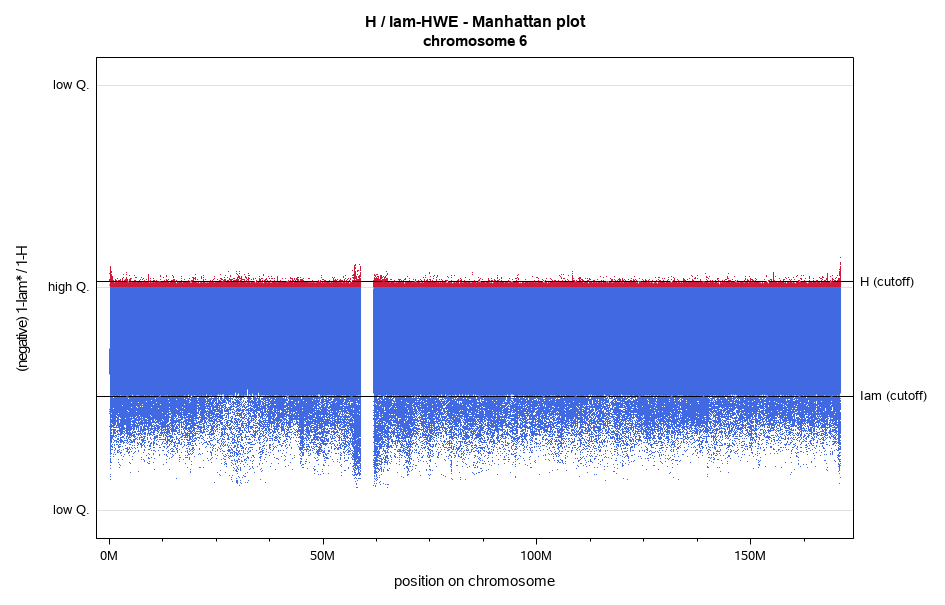

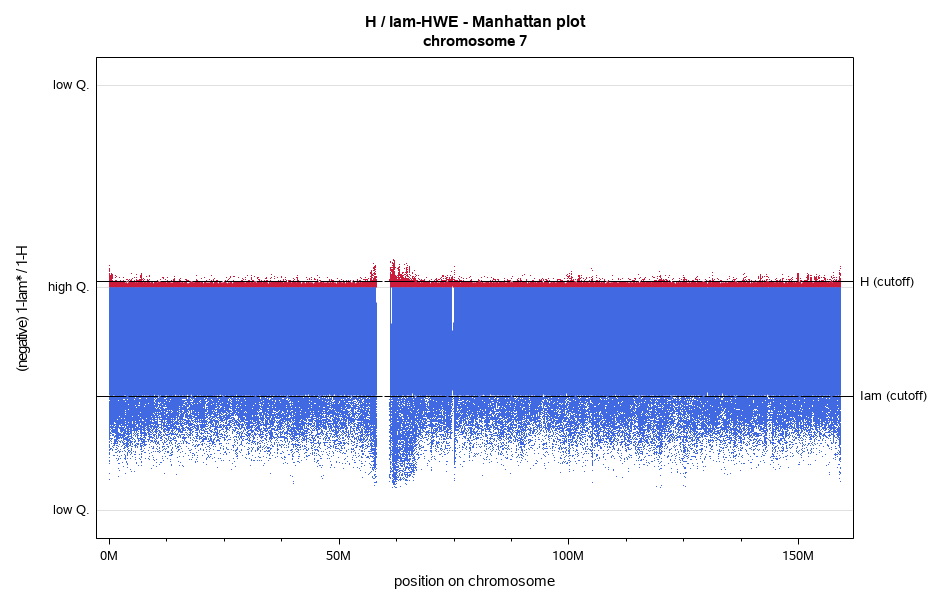

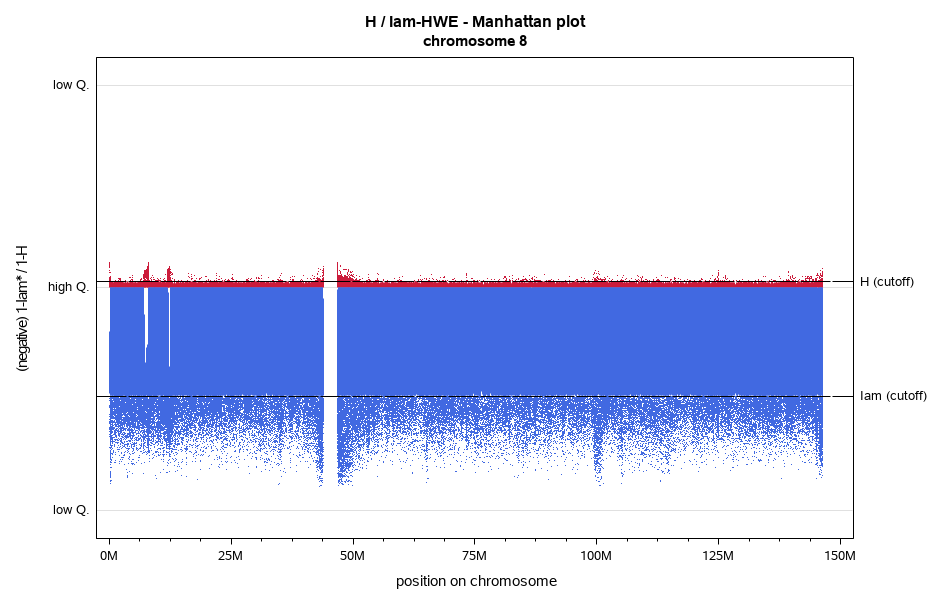


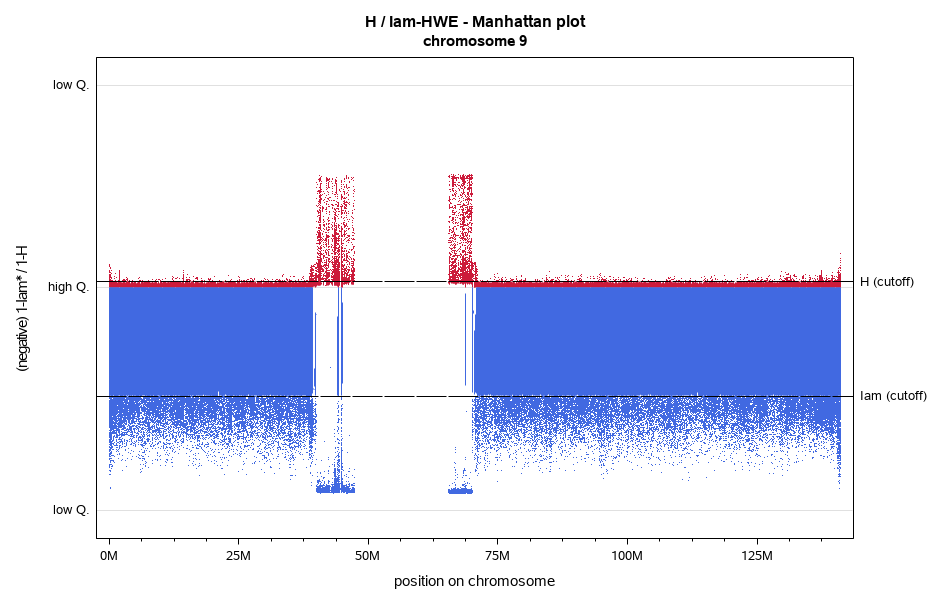

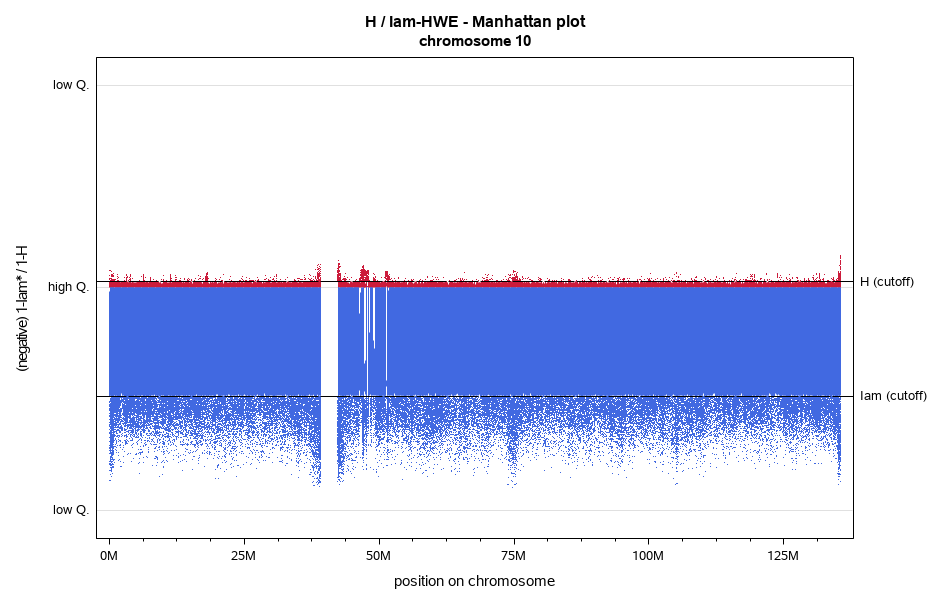

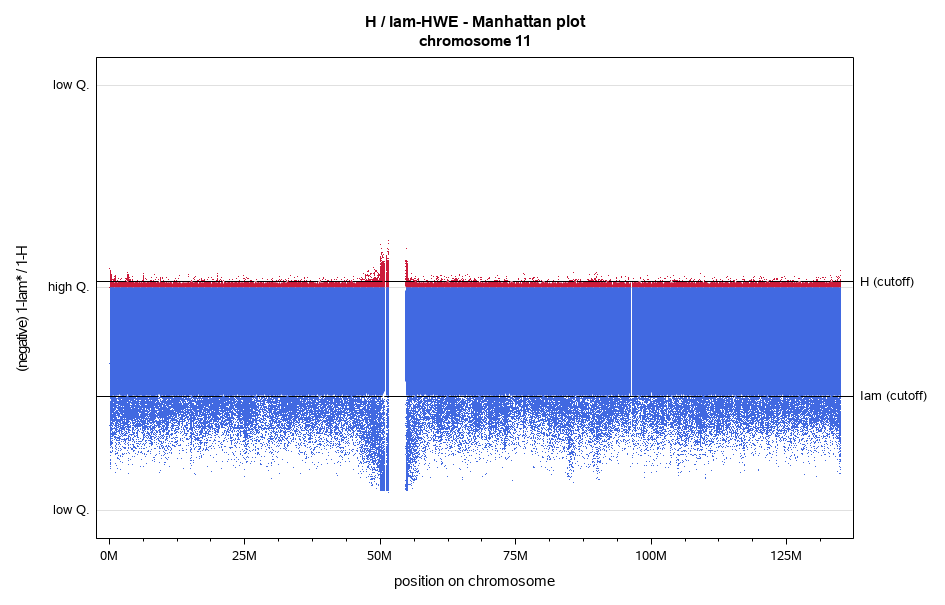

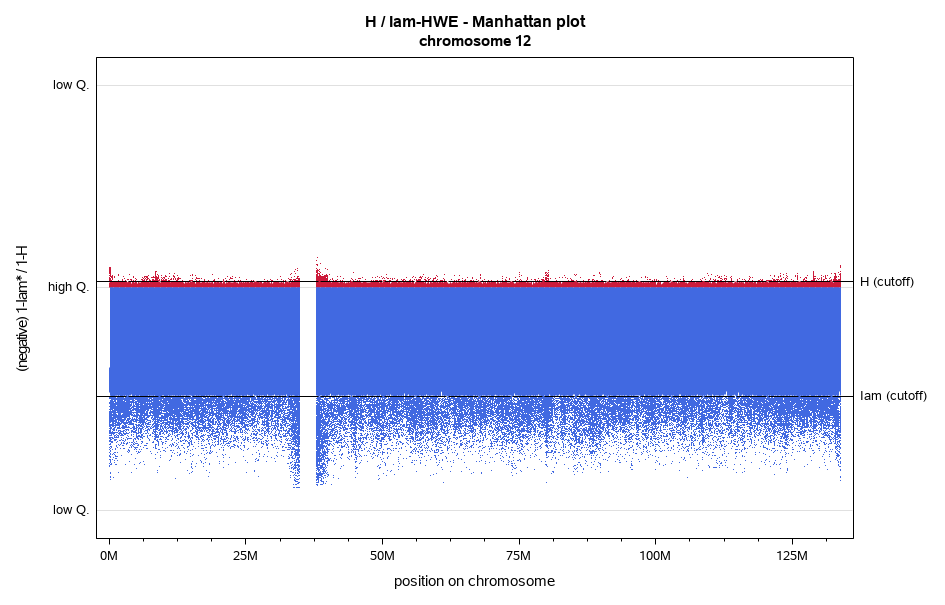

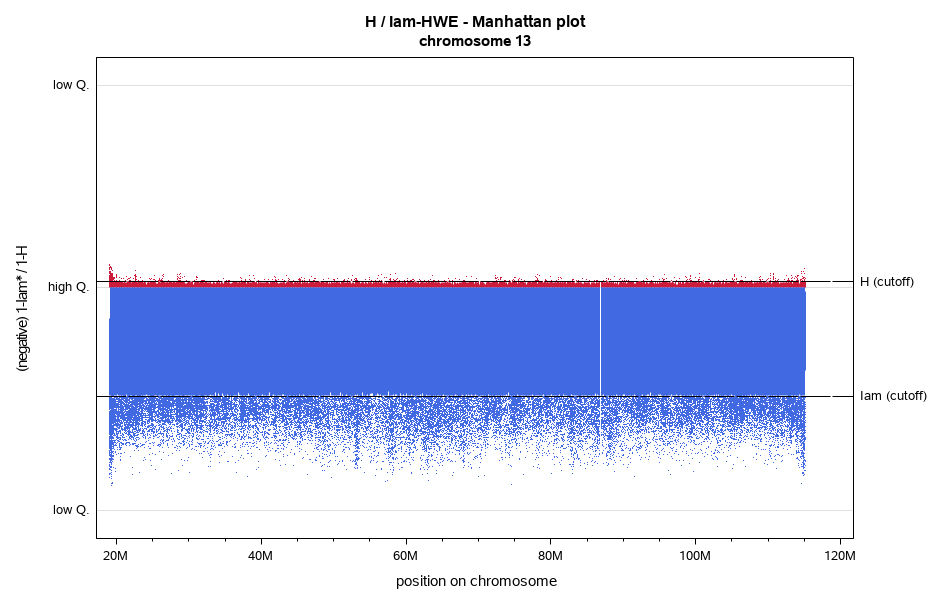

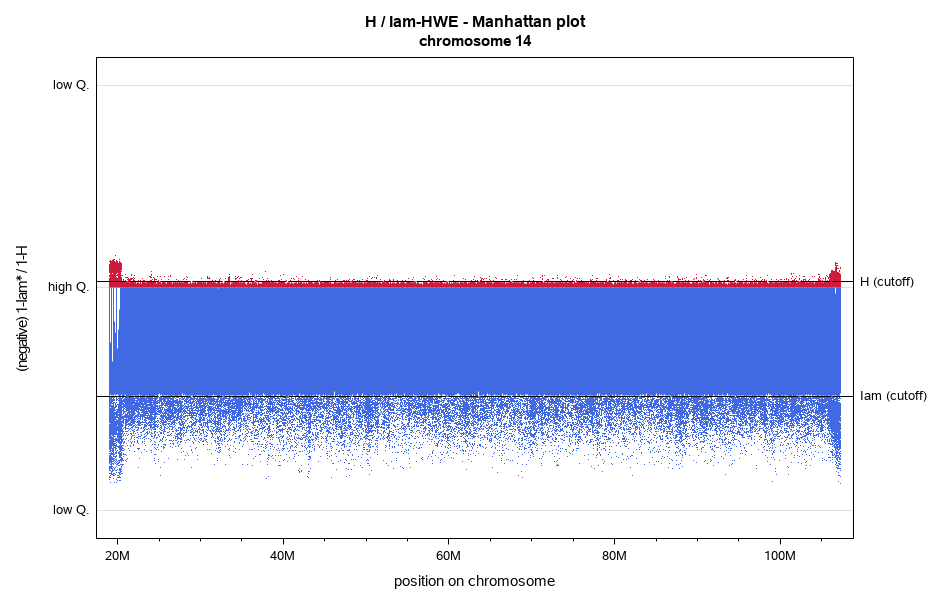

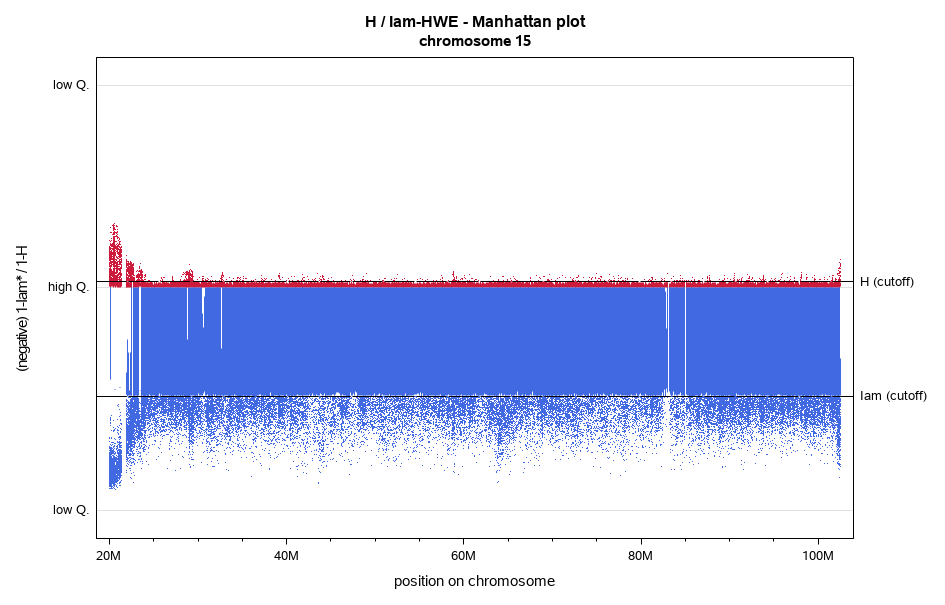

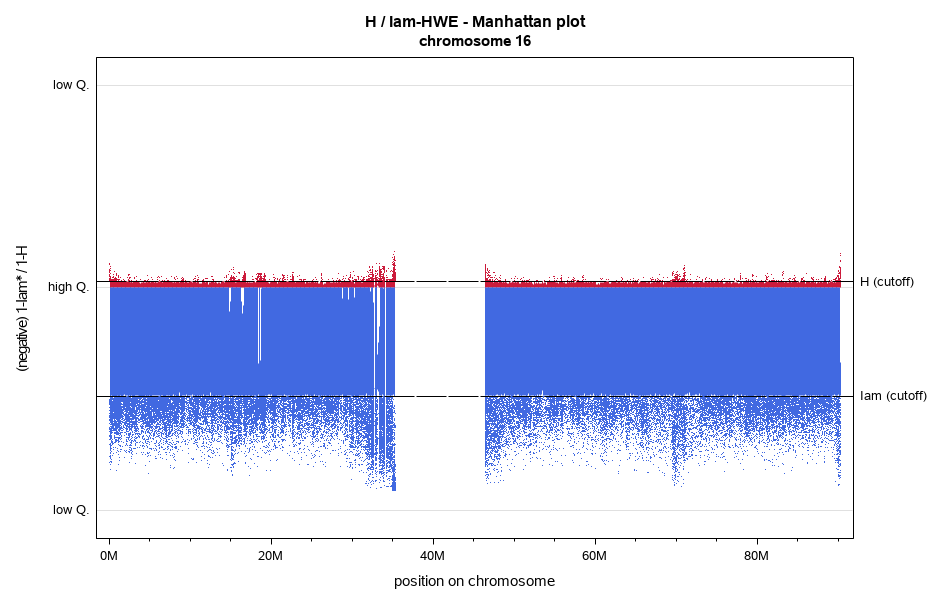


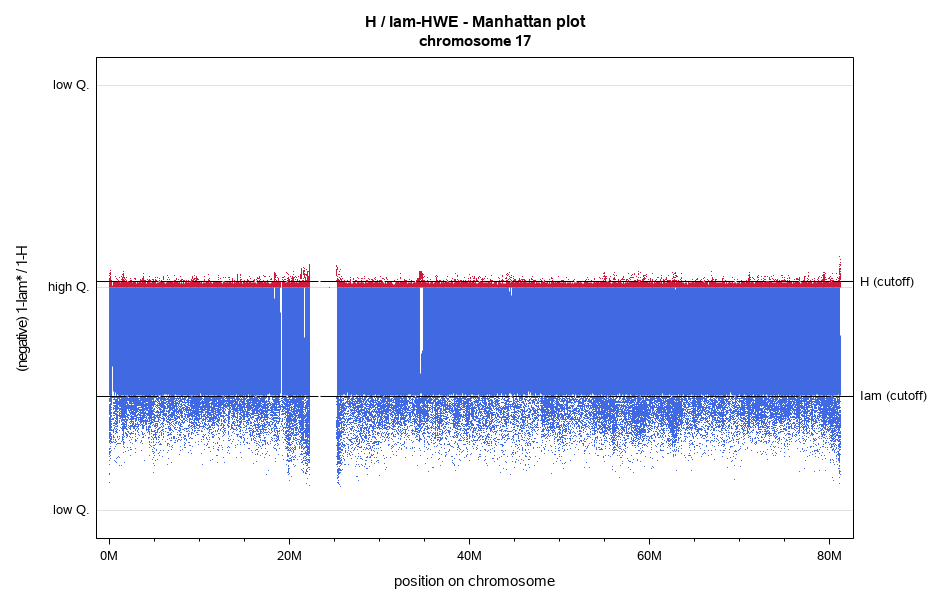

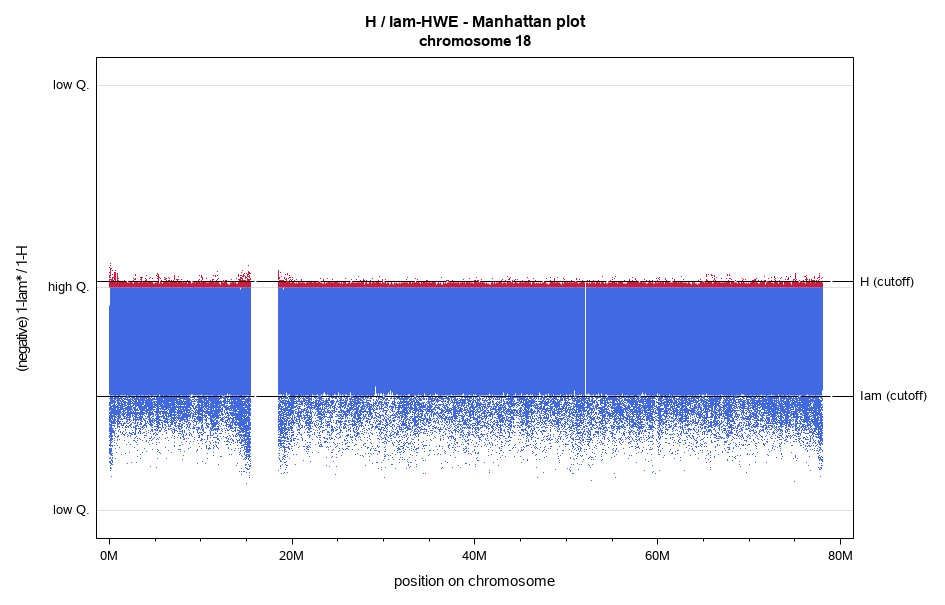

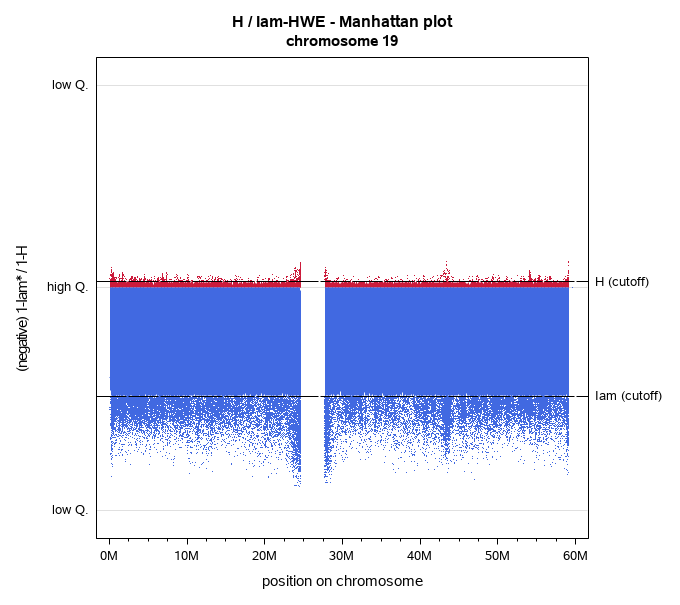

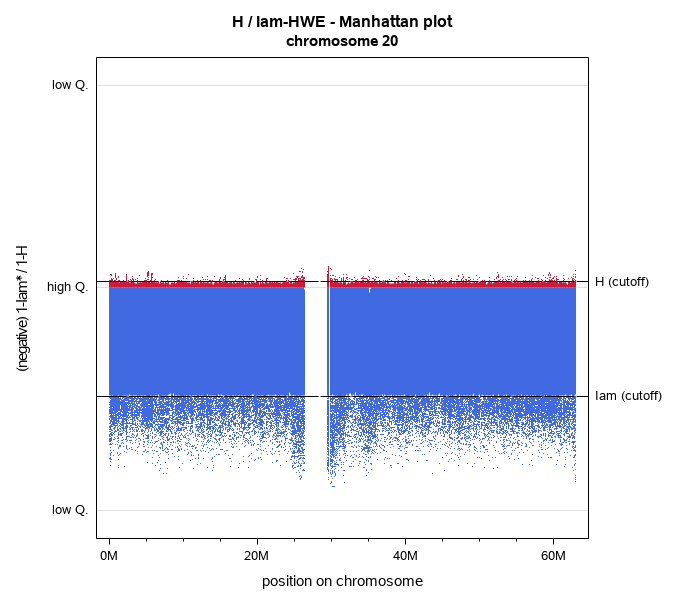

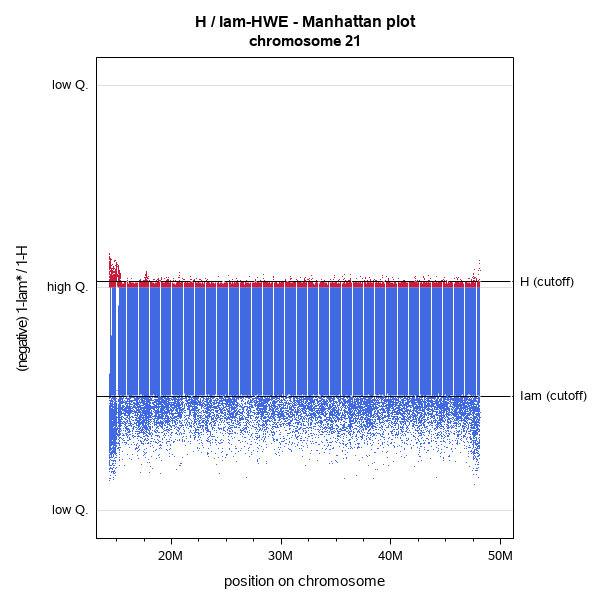

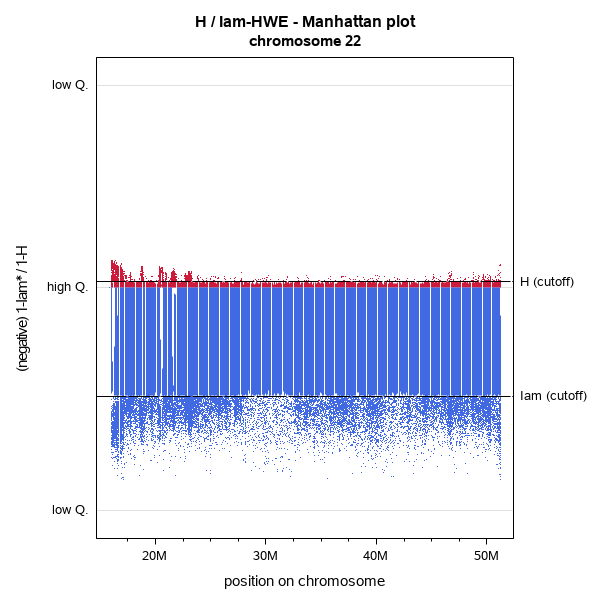


# Comparing *Iam* with *info*

Additional file: Figure S2 Comparison: *Iam* with *info* (Impute2)


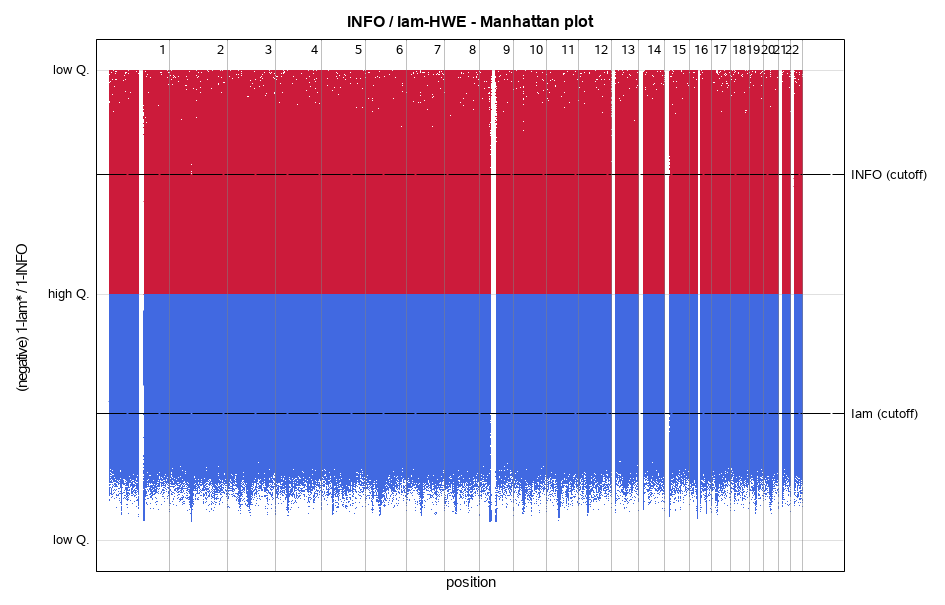


Upper panel: info (low Q.: info=0; high Q.: info=1; Thresholds info (cutoff=0.8) displayed as a power of 3.38 so that the thresholds are visually comparable; lower panel: ${Iam}_{HWE}$(low Q.: ${Iam}_{HWE}$=0; high Q.: ${Iam}_{HWE}$=1; Thresholds *Iam*cutoff=0.47): the threshold for ${Iam}_{HWE}$was defined according a robust 99.9999999% random interval (assuming a two-dimensional normal distribution)

Additional file: Figure S3 Comparison: *Iam* with *info* (Impute2): chromosome 1


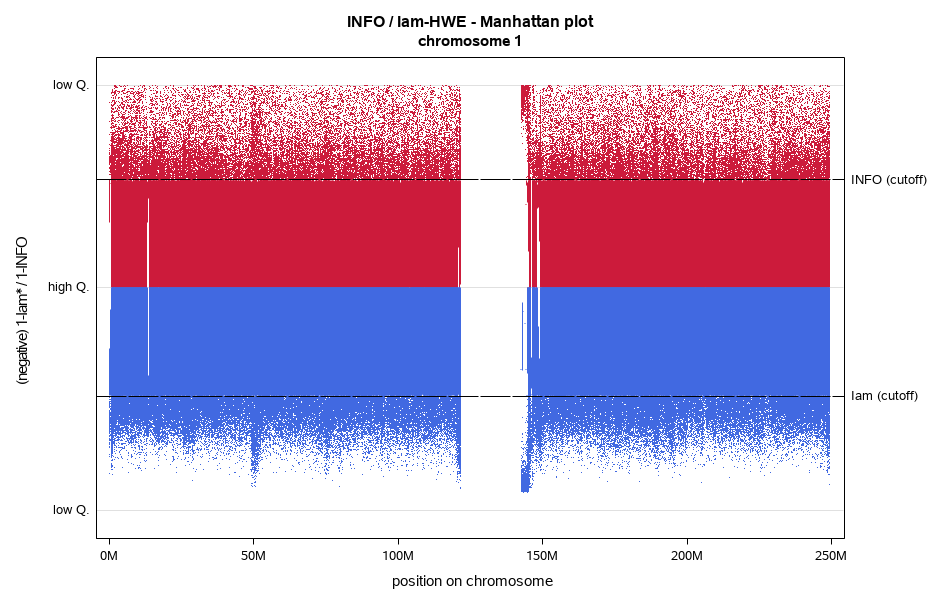


Upper panel: info (low Q.: *info*=0; high Q.: *info*=1; Thresholds *info*(cutoff=0.8) displayed as a power of 3.38 so that the thresholds are visually comparable; lower panel: ${Iam}_{HWE}$(low Q.: ${Iam}_{HWE}$=0; high Q.: ${Iam}_{HWE}$=1; Thresholds *Iam* cutoff=0.47): the threshold for ${Iam}_{HWE}$was defined according a robust 99.9999999% random interval (assuming a two-dimensional normal distribution)
