# Supplementary material for: Iam hiQ—a novel pair of accuracy indices for imputed genotypes
Source: BMC Bioinformatics. 2022 Jan 24;23:50. doi: 10.1186/s12859-022-04568-3 (PMC8785528; doi:10.1186/s12859-022-04568-3)
Supplement: Supplementary file 2 — Additional file 2. Other members (not co-authors) of the International Lung Cancer Consortium (ILCCO). [file 12859_2022_4568_MOESM2_ESM.pdf]

Other members of the International Lung Cancer Consortium (ILCCO)

Hidemi Ito [38], Wang Ying [39], Michael J. Thun [40] , Leal Letícia Ferro [41], Rui Manual Reis [41], Margaret Spitz [42], Hormuzd Katki [43] , Iona Cheng [44], Pascal Guenel [45], Therese Truong [45], Monica Neri [46], Ann Tjonneland [47], Golorva Olga [48], Jiang Gui [49], Thorunn Rafnar [50], Jolanta Lissowska [51], Hongbin Shen [52], Summer S. Han [53], Eleonora Fabianova [54], Vladimir Janout [55], Li-Shiun Chen [56], Lenka Foretova [57], Keitaro Matsuo [58], Hermann Brenner [59], Qingyi Wei [60], Paolo Vineis [61], Tommaso A. Dragani [62], Francesca Colombo [62], Dario Consonni [62], Jie Zhang [63], James McKay [64], Irene Bruske [65], Brian Cox [66], Marc Chadeau [67], Simone Benhamou [68], David Zaridze [69], Ivana Holcatova [70], Vladimir Benko [70], Michele Cote [71], Kouya Shiraishi [72], Chikako Kiyohara [73], Yohan Bossé [74], Ping Yang [75], Irene Orlow [76], Marjorie Zauderer [77], Paolo Boffetta [78], Takashi Kohno [79], Michael Alavanja [79], Nat Rothman [79], Brid Ryan [79], Curtis C. Harris [79], Jiyeon Choi [79], Daniela Seminara [79], Qin Lan [79], Maki Inoue-Choi [79], Chao Hsiung [80], Peter Rudnai [81], Dana Mates [82], Joshua E. Muscat [83], John R. McLaughlin [84], Adeline Seow [85], Richard S. Houlston [86], Yun-Chul Hong [87], Jose Ignacio Mayordomo [88], Bonnie E. Gould Rothberg [89], Hamideh Rashidian [90], Kazem Zendehdel [90], Beata Swiatkowska [91], Ugo Pastorino [92], Zuo-Feng Zhang [93], Angela Pesatori [94], John Wiencke [95], Lani Park [96], Hal Moregenstern [97], Brenda Diergaarde [98], Jian-Min Yuan [98], Juan Miguel Barros-Dios [99], Alberto Ruano Ravina [99], Ann Schwartz [100]

38. Aichi Cancer Center, Japan
39. American Cancer Society, Atlanta, GA, USA
40. American Cancer Society, Atlanta, GA, USA
41. Barretos Cancer Hospital, Molecular Oncology Research Center, Brasil
42. Baylor College of Medicine, Houston, Texas, USA
43. Biostatistics Branch, Division of Cancer Epidemiology and Genetics, National Cancer Institute, NIH, USA
44. Cancer Prevention Institute of California, Fremont, CA, USA
45. CESP – Cancer and Environment, Villejuif Cedex, France
46. Clinical and Molecular Epidemiology, IRCCS San Raffaele Pisana, Rome, Italy
47. Danish Cancer Society, Denmark
48. Dartmouth College, Hanover NH, USA
49. Dartmouth College, Lebanon, NH, USA
50. deCODE Genetics, Division of Cancer, Reykjavik, Iceland
51. Department of Cancer Epidemiology and Prevention, Cancer Center and Maria Sklodowska-Curie Institute of Oncology, Warsaw, Poland
52. Department of Epidemiology & Biostatistics, Nanjing Medical University School of Public Health, Nanjing, China
53. Department of Medicine, Stanford University School of Medicine, Stanford University, Stanford, CA, USA
54. Department of Occupational Health, Specialized State Health Institute, Banska Bystrica, Slovakia
55. Department of Preventive Medicine, Palacky University Faculty of Medicine, Olomouc, Czech Republic
56. Department of Psychiatry, St. Louis University, St. Louis, MO,USA
57. Dept Cancer Epidemiology & Genetics, Masaryk Memorial Cancer Institute, Brno, Czech Republic
58. Division of Molecular Medicine, Aichi Cancer Center Research Institute, Nagoya, Japan
59. DKFZ, Germany
60. Duke University, Durham, NC, USA
61. Environmental Epidemiology, Division of Epidemiology, Public Health and Primary Care, Imperial College London, UK
62. Fondazione IRCCS Istituto Nazionale Tumori, Milan, Italy
63. Fudan University Shanghai Cancer Center, Shanghai, China
64. Genetic Cancer Susceptibility, International Agency for Research on Cancer, Lyon, France
65. Helmholtz Zentrum München, Germany
66. Hugh Adam Cancer Epidemiology Unit, Department of Preventive and Social Medicine, Dunedin School of Medicine, University of Otago, Dunedin, New Zealand
67. Imperial College London; UK
68. INSERM, Unit 346, Fondation Jean Dausset, CEPH, Paris, France
69. Institute of Carcinogenesis, Department of Epidemiology and Prevention, Russian N.N. Blokhin Cancer Research Centre, Moscow, Russia
70. Institute of Hygiene and Epidemiology, Charles University, First Faculty of Medicine, Czech Republic
71. Karmanos Cancer Institute, Detroit, MI, USA
72. Kitasato University, Japan
73. Kyushu University, Department of Preventive Medicine, Graduate School of Medical Sciences, Fukuoka, Japan
74. Laval University, Department of Molecular Medicine, Institut universitaire de cardiologie et de pneumologie de Québe, CA
75. Mayo Clinic Cancer Center, Rochester, MN, USA
76. Memorial Sloan-Kettering Cancer Center, NY; USA
77. Memorial Sloan-Kettering Cancer Center, NY; USA
78. Mount Sinai School of Medicine, NY, USA

79. National Cancer Center Research Institute, Japan
80. National Health Research Institutes, Taiwan
81. National Institute of Environmental Health, Fodor József National Center for Public Health, Budapest, Hungary
82. Occupational Health Department, Institute of Public Health, Bucharest, Romania
83. Penn State College of Medicine, Penn State Cancer Institute, Division of Population, Hershey, PA, USA
84. Samuel Lunenfeld Research Institute, Mount Sinai Hospital, Toronto, Canada
85. Saw Swee Hock School of Public Health, National University of Singapore, Singapore
86. Section of Cancer Genetics, Institute of Cancer Research, Sutton, Surrey, UK
87. Seoul National University, College of Medicine, Dept. of Preventive Medicine, Seoul, Korea
88. Servicio de Oncología Médica, Hospital Clínico Universitario, Zaragoza, Spain
89. Smilow Cancer Hospital Oncology Extended Care Clinic, Yale Cancer Center, Yale School of Medicine, New Haven, CT, USA
90. Tehran University of Medical Sciences, Cancer Institute of Iran, Tehran, Iran
91. The Reference Center for Asbestos Exposure & Health Risk Assessment, Nofer Institute of Occupational Medicine, Lódz, Poland
92. Thoracic Surgery Unit, National Cancer Institute, Milan, Italy
93. UCLA School of Public Health, Department of Epidemiology, Los Angeles, CA, USA
94. Universit degli Studi di Milano; Milano, Italy
95. University of California, Dept. of Epidemiology and Biostatistics, San Francisco, CA, USA
96. University of Hawaii Cancer Center, University of Hawaii at Manoa , Honolulu, HI, USA
97. University of Michigan, MI, USA
98. University of Pittsburg, Pittsburg; PA, USA
99. University of Santiago de Compostela, Santiago de Compostela, Spain
100. Wayne State University School of Medicine, Detroit, MI, USA
